# A *PLAG1* mutation contributed to stature recovery in modern cattle

**DOI:** 10.1038/s41598-017-17127-1

**Published:** 2017-12-07

**Authors:** Yuri Tani Utsunomiya, Marco Milanesi, Adam Taiti Harth Utsunomiya, Rafaela Beatriz Pintor Torrecilha, Eui-Soo Kim, Márcio Silva Costa, Tamíris Sayuri Aguiar, Steven Schroeder, Adriana Santana do Carmo, Roberto Carvalheiro, Haroldo Henrique Rezende Neves, Romulo Cláudio Morozini Padula, Thayla Souza Sussai, Ludmilla Balbo Zavarez, Rafael Silva Cipriano, Maria Margareth Theodoro Caminhas, George Hambrecht, Licia Colli, Elisa Eufemi, Paolo Ajmone-Marsan, Deneb Cesana, Marco Sannazaro, Maurizio Buora, Michele Morgante, George Liu, Derek Bickhart, Curtis Paul Van Tassell, Johann Sölkner, Tad Stewart Sonstegard, José Fernando Garcia

**Affiliations:** 1 São Paulo State University (Unesp). School of Agricultural and Veterinarian Sciences, Jaboticabal. Department of Preventive Veterinary Medicine and Animal Reproduction, São Paulo, Brazil; 2 International Atomic Energy Agency (IAEA) Collaborating Centre on Animal Genomics and Bioinformatics, Araçatuba, São Paulo, Brazil; 3 Istituto di Zootecnica and BioDNA Centro di Ricerca sulla Biodiversità e sul DNA Antico, Università Cattolica del Sacro Cuore, Piacenza, Italy; 4 São Paulo State University (Unesp). School of Veterinary Medicine, Araçatuba. Department of Support, Production and Animal Health, São Paulo, Brazil; 5 Recombinetics, Inc., St Paul, MN 55104 USA; 6 UFPI – Universidade Federal do Piauí, Piauí, Brazil; 7 Animal Genomics and Improvement Laboratory, Agricultural Research Service, USDA, Beltsville, Maryland USA; 8 Escola de Veterinária e Zootecnia (EVZ), Universidade Federal de Goiás - UFG, Campus Samambaia, Goiânia, Goiás Brazil; 9 São Paulo State University (Unesp). School of Agricultural and Veterinarian Sciences, Jaboticabal. Department of Animal Science, São Paulo, Brazil; 10 GenSys Consultores Associados, Porto Alegre, Brazil; 11 Centro Universitário Católico Salesiano, Araçatuba, São Paulo, Brazil; 12 Department of Anthropology, University of Maryland, College Park, USA; 13 Dipartimento di Storia, Archeologia e Storia dell’Arte, Facoltà di Lettere e Filosofia, Università Cattolica del Sacro Cuore, Milano, Italy; 14 Società Friulana di Archeologia, Udine, Italy; 15 Istituto di Genomica Applicata, Udine, Italy; 16 Dipartamento di Scienze Agrarie ed Ambientali, Università di Udine, Udine, Italy; 17 BOKU - University of Natural Resources and Life Sciences, Department of Sustainable Agricultural Systems, Division of Livestock Sciences, Vienna, Austria

## Abstract

The recent evolution of cattle is marked by fluctuations in body size. Height in the *Bos taurus* lineage was reduced by a factor of ~1.5 from the Neolithic to the Middle Ages, and increased again only during the Early Modern Ages. Using haplotype analysis, we found evidence that the bovine *PLAG1* mutation (*Q*) with major effects on body size, weight and reproduction is a >1,000 years old derived allele that increased rapidly in frequency in Northwestern European *B. taurus* between the 16^th^ and 18^th^ centuries. Towards the 19^th^ and 20^th^ centuries, *Q* was introgressed into non-European *B. taurus* and *Bos indicus* breeds. These data implicate a major role of *Q* in recent changes in body size in modern cattle, and represent one of the first examples of a genomic sweep in livestock that was driven by selection on a complex trait.

## Introduction

**Figure 1:**
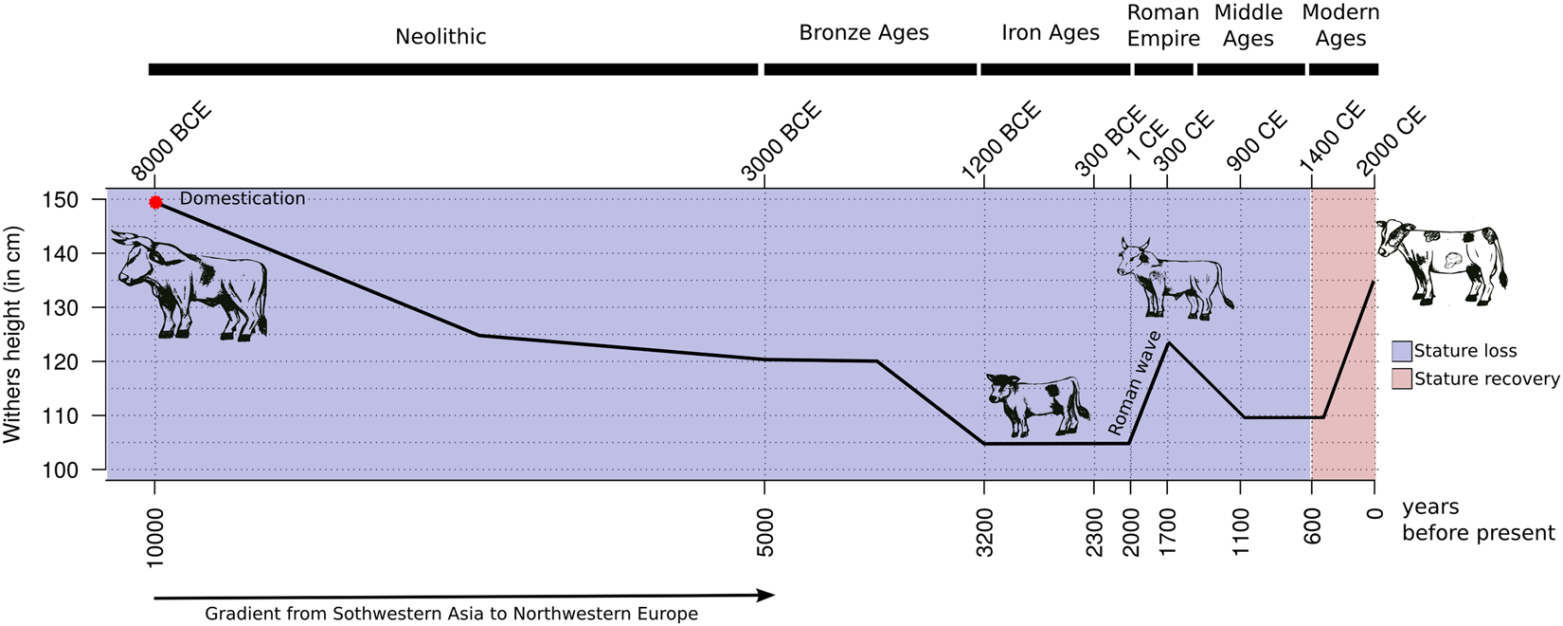
Schematic of clinal and temporal variation in cattle stature. The line art was produced in R v.3.3.2^74^ and enhanced in Inkscape v0.48.4-r9939^75^.

The extinct wild auroch (*Bos primigenius*) lost stature during late Pleistocene, decreasing from a withers height range of 165–185 cm to 145–160 cm^1^. Between 280,000^2^ and 330,000^3^ years before present (yBP), the ancestral auroch population diverged into two distinct lineages that would later originate the humpless *Bos taurus* and the humped *Bos indicus* cattle. Towards the beginning of the Holocene, *B. taurus* and *B. indicus* were independently domesticated in the Fertile Crescent (~10,500 yBP) and in the Indus Valley (~8,500 yBP), respectively^4,5^. Later, *B. taurus* suffered a further decline in stature between the Neolithic and the Early Middle Ages^6^, approaching wither sizes of 95–123 cm^1^. Archaeological data further suggested that stature loss followed a gradient from Southwestern Asia towards Northwestern Europe that coincides with the post-domestication route of Europe colonization^7^. A counterpoint to this process was also suggested, namely introduction of cattle in Northern and Western Europe by the Roman Empire around the 1^st^ century that were much larger than Celtic or Germanic cattle, ranging from 105 to 142 cm^1^. However, stature of Northwestern cattle decreased again shortly after the fall of the Roman Empire. Along with the intensification of artificial selection in the past few centuries, *B. taurus* entered a process of stature recovery that started between the 15^th^ and 18^th^ centuries and that lasted until recently^1,6^. Consequently, modern European breeds typically range from 105 to 155 cm in average withers height^8^, which represents a 1.10- to 1.26-fold increase in stature compared to the Early Middle Ages (Fig. 1).

If major genetic variants contributed to stature recovery since the Early Modern Ages, their selection signatures should be detectable from genomic data of modern cattle breeds. Randhawa *et al*.^9^ contrasted genome-wide single nucleotide polymorphism (SNP) data of European *B. taurus* breeds with high (145–155 cm) and low (105–133 cm) median withers height and found that, relative to the UMD v3.1 genome assembly^10^, the most significant signature related to stature mapped to chromosome 14 (CHR14) positions 24.79–28.25 million base-pairs (Mbp). This signature has been recently confirmed with whole genome sequence data of four *B. taurus* breeds and constrained to a smaller region spanning positions 24.80–25.08 Mbp^11^, where the pleiomorphic adenoma gene 1 (*PLAG1*) is located. Nevertheless, it is still unclear whether this selective sweep is traceable back to the period between the 15^th^ and 18^th^ centuries.

Following the milestone publication by Karim *et al*.^12^, we and others^13–22^ have previously reported that this CHR14 region is also a major pleiotropic quantitative trait locus (QTL) affecting cattle body size and reproduction, supporting an important role of *PLAG1* in recent changes in stature in cattle. The candidacy of *PLAG1* is further supported by functional evidence, since the transcription factor encoded by this gene regulates the expression of insulin-like growth factors^14,23–26^. Moreover, mice carrying a null *PLAG1* allele exhibit growth retardation and reduced fertility^27^. An intriguing observation however is that the QTL is detectable in modern breeds of both *B. taurus* and *B. indicus* cattle, which is unexpected given the long divergence time between these two subspecies.

Three main hypotheses are consistent with a major stature QTL that is detectable in both *B. taurus* and *B. indicus*: (i) variants that were present in the ancestral *B. primigenius* population still segregate in both subspecies; (ii) separate derived alleles account for the QTL in the two subspecies; or (iii) lineage-specific mutations have been recently introgressed from one subspecies to the other. Together with the evidence of selection, hypothesis (i) would imply that very ancient (>280,000 years old) standing neutral variants at the *PLAG1* locus became differentially advantageous under certain circumstances. This hypothesis could be confirmed by showing that ancestral haplotypes have positive effects on body size in both subspecies. On the other hand, hypotheses (ii) and (iii) would require derived haplotypes, and could be tested by showing that *B. taurus* and *B. indicus* either carry different (ii) or identical (iii) derived haplotypes positively increasing body size.

Here, we aimed at elucidating the patterns of gene flow of *PLAG1* haplotypes by gathering evidence from the *B. indicus* lineage and contrasting against data of worldwide *B. taurus* breeds. It was our objective to investigate whether the selective sweep surrounding the haplotype was traceable to the recent event of stature recovery in Northwestern Europe.

## Results

### Association analysis in *B. indicus* maps a derived haplotype tagging the *PLAG1* mutation (*Q*)

In support to the hypothesis of introgression, Fortes *et al*.^14^ presented evidence that the QTL haplotype associated with increased body size has an exclusive *B. taurus* origin in Brahman, a breed with average 91% *B. indicus* and 9% *B. taurus* ancestry^28^. Although Brahman could be an exception given its higher levels of *B. taurus* ancestry in comparison to breeds that are generally considered purebred *B. indicus*, similar heterogeneity of haplotype origin at this QTL in other breeds is plausible since most of the modern *B. indicus* populations were recently subjected to *B. taurus* introgression^29–31^.

In order to test whether the introgression hypothesis holds in other *B. indicus* populations, we decided to re-map the QTL in Brazilian Nellore cattle. Our past investigation in this breed^18,19,22^ pointed to a haplotype with positive effects on weight and conformation traits, in agreement with the findings by Fortes *et al*.^14^. However, as for other *B. indicus* breeds, a *B. taurus* origin for this haplotype in Nellore is less clear since we found that the current population has on average only 0.9% *B. taurus* ancestry^28,31^. Nevertheless, the introgression hypothesis remains plausible given the documented crossbreeding in Brazil between *B. taurus* brought by colonizers and *B*. *indicus* imported from India in the late 19^th^ century and early 20^th^ century^6^. Moreover, considering a genome size of ~2.87 billion base-pairs (Gbp) with ~22,000 protein coding genes^32^, 0.9% would correspond to ~25.8 Mbp of sequence, which is approximately one fourth of an average-sized chromosome (~100 Mbp) or ~200 protein-coding genes.

We started by analyzing Illumina® BovineHD (HD) genotypes of a sample of 779 Nellore bulls with deregressed estimated breeding values (dEBVs) for birth weight obtained from records of 846,782 calves. These bulls sired over ten Nellore generations and comprised animals born between 1965 and 2008, with 90% born after 1990 and 50% after 2000. We selected birth weight as a proxy for body size because: (i) withers height measurements were not available for these animals; (ii) the QTL was associated with differences in fetal expression of *PLAG1* in *B. taurus* ^12^; and (iii) birth weight is moderately heritable (*h* ^2^ = 0.37) and affected by fewer environmental effects in comparison to other massively recorded traits in Nellore cattle. Whole chromosome haplotypes were constructed from 15,132 SNP markers on CHR14 with a Hidden Markov Model^33^. Haplotypes at shorter segments were then determined in order to map the QTL via regression analysis.

**Figure 2:**
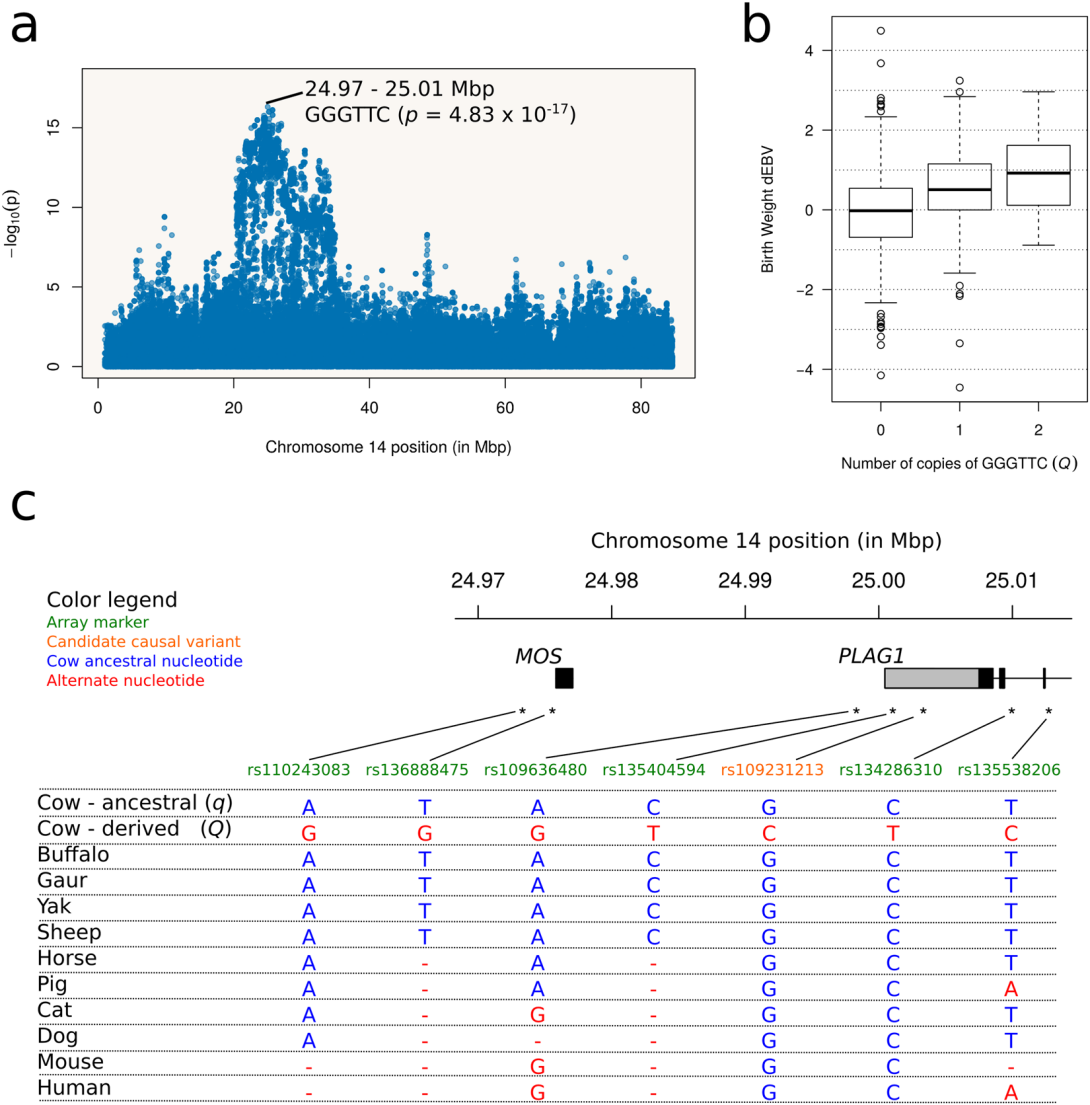
Identification of a haplotype tagging the *PLAG1* mutation (*Q*) in *B. indicus*. (**a**) Scatterplot showing the birth weight (dEBV) haplotype association mapping on chromosome 14 in Nellore cattle. Maximum association (*p* = 4.83 × 10^−17^) was detected in a ~39.5 kbp segment spanning positions 24973324 to 25012733, where *MOS* and the 3′ end of *PLAG1* are located. (**b**) The distribution of birth weight dEBVs according to number of copies of the tag haplotype indicates an additive effect of 0.311 ± 0.037 kg. (**c**) Orthology analysis suggesting *Q* to be a derived mutation.

We were able to map associations to a ~39.5 thousand base-pairs (kbp) haplotype (*p* = 4.83 × 10^−17^) spanning positions CHR14:24973324–25012733 (Fig. 2a). Importantly, this segment was a subset of the ~271 kbp region reported by Boitard *et al*.^11^ in their selection signature analysis using *B. taurus* sequence data. This region contained the v-mos Moloney murine sarcoma viral oncogene homolog (*MOS*) and a portion of the 3′ end of *PLAG1*. The significant haplotype included nucleotides G – rs110243083, G – rs136888475, G – rs109636480, T – rs135404594, T – rs134286310 and C – rs135538206, and will be hereafter denoted GGGTTC. Marker rs135404594 was located in the 3′-UTR of *PLAG1*, whereas markers rs134286310 and rs135538206 were intronic to *PLAG1*. All remaining markers in the haplotype were intergenic. Frequency of GGGTTC was 17.8%, with an estimated effect of 0.311 ± 0.037 kg on birth weight dEBVs. The distribution of dEBVs according to number of copies of GGGTTC followed an additive pattern (Fig. 2b), confirming previous reports^12,18^.

For the sake of simplicity, we will denote *Q* the unknown causal allele with positive effect on birth weight, whereas *q* will refer to the alternative allele, following notation introduced by Karim *et al*.^12^. Assuming *Q* co-segregates with the GGGTTC tag in Nellore, 18 (2.3%), 241 (30.9%) and 520 (66.8%) bulls had predicted genotypes *QQ*, *Qq* and *qq*, respectively, which did not significantly deviate from Hardy-Weinberg Equilibrium (HWE, *p* = 0.215). Among the six alternative haplotypes, the most common was ATACCT (70.4%). An orthology analysis revealed ATACCT to be the ancestral haplotype (Fig. 2c). Therefore, *q* and *Q* were most likely the ancestral and derived alleles, respectively, in agreement with Fortes *et al*.^14^. Consequently, the data provided little support for the hypothesis of *Q* being a mutation already present in the population of aurochsen ancestral to both *B. taurus* and *B. indicus* cattle. Furthermore, the existence of other low frequency haplotypes in this sample suggested that *q* occurs in several different haplotype backgrounds, consistent with an ancestral allele.

### Ancestry and sequence analysis in *B. indicus* reveals *B*. *taurus* introgression

**Figure 3:**
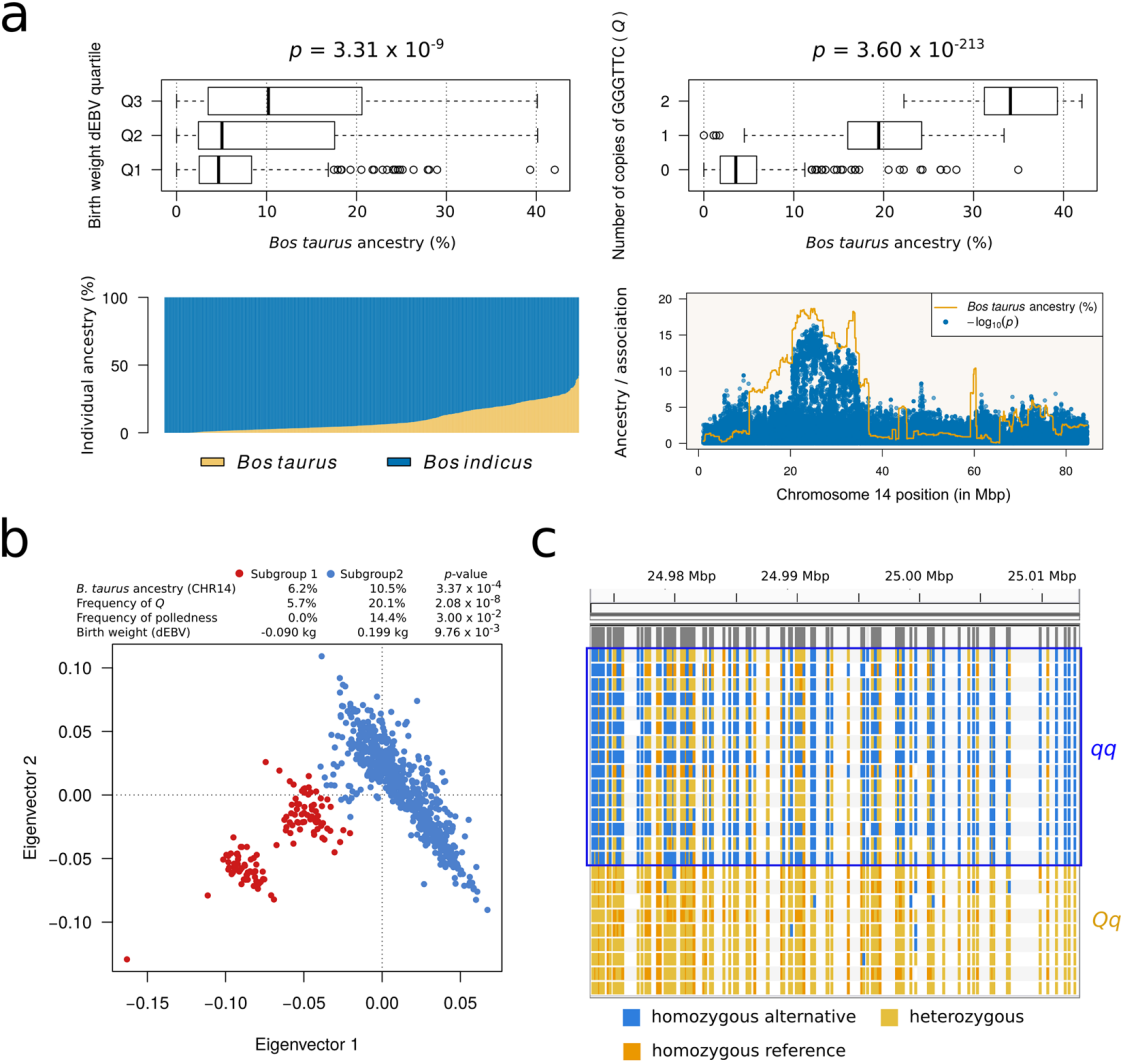
*B. taurus* introgression as a source for *Q* in *B. indicus*. (**a**) Ancestry analysis showing that both birth weight dEBVs and occurrence of *Q* were positively associated with percentage of *B. taurus* ancestry on chromosome 14. (**b**) A principal components analysis revealed two breeding subgroups of Nellore cattle differing in *B. taurus* ancestry, frequency of *Q*, incidence of polledness (presumably also resulting from *B. taurus* introgression) and mean of birth weight dEBV. (**c**) Next-generation sequencing data of 24 Nellore bulls confirming a *B. taurus* origin of *Q* in *B. indicus*. Each row represents a bull, and colored vertical bars represent genotypes at different variant sites.

In order to test the hypothesis of two separate derived mutations in *B. taurus* and *B. indicus*, we searched for carriers of *Q* among Nellore bulls that directly descended from animals imported from India in the 20^th^ century and that therefore were unlikely to carry *B. taurus* ancestry. Our sample contained 15 animals meeting this criterion. Given the variant was under HWE, four to five heterozygous bulls were expected assuming a heterozygosity range of 27.6–34.2% (95% CI considering a standard error of 1.7%) but all 15 were predicted to be *qq*. In fact, the earliest predicted carrier of *Q* was a polled animal born in 1975. Coincidently, the polled allele in Brazilian Nellore^34^ is deemed to be of *B. taurus* origin. We further performed a model-based clustering analysis^35^ to estimate *B. taurus* ancestry on CHR14 using a panel of reference breeds^36,37^. Estimated percentages ranged from 0.0% to 46.9% (mean = 10.0 ± 9.55%) and were positively associated with birth weight dEBVs (*p* = 3.31 × 10^−9^) and number of copies of GGGTTC (*r* = 0.845, *p* = 3.60 × 10^−213^), indicating a *B. taurus* origin for *Q* in the *B. indicus* lineage (Fig. 3a). Refinement of ancestry estimates at the level of individual loci revealed that the recombination break points of introgressed segments mimicked the topology of the haplotype-based association analysis, suggesting that birth weight associations on this chromosome were essentially driven by haplotypes of *B. taurus* origin. The coordinate CHR14:24459302-25246448 exhibited the highest *B. taurus* introgression, which was estimated at 18.64% across individuals. Remarkably, this ~787.2 kbp segment contained the ~39.5 kbp region identified earlier through association analysis, and its ancestry estimate was very close to the frequency of the GGGTTC haplotype.

As genetic stratification into two breeding subgroups has been reported in the Nellore breed^18,38^, we decided to investigate whether *B. taurus* ancestry on CHR14, frequency of *Q*, mean birth weight dEBV and frequency of polledness further differed between these subgroups. Subgroup 1 was known to be selected for production traits, whereas subgroup 2 has been selected mainly for breed type. Also, breeders of subgroup 1 aim at average birth weight in order to maintain positive genetic trends for calving ease. We were able to retrieve horned/polled phenotypes for a subset of 379 bulls and assign individuals to breeding subgroups via a k-means clustering algorithm^39^. In comparison with subgroup 1, subgroup 2 presented larger average *B. taurus* ancestry on CHR14 (10.5% against 6.2%, *p* = 3.37 × 10^−4^), higher frequency of *Q* (20.1% against 5.7%, *p* = 2.08 × 10^−8^), higher incidence of polledness (14.4% against 0.0%, *p* = 0.03), and larger mean birth weight dEBV (0.199 kg against −0.090 kg, *p* = 9.76 × 10^−3^) (Fig. 3b). These results further supported the positive effect of *Q* on weight and a *B. taurus* origin for both *Q* and polledness in Nellore cattle.

Next, we sequenced the whole genomes of 24 Nellore bulls at ~9x coverage. Nine of these bulls were predicted to be *Qq* and fifteen were predicted to be *qq* based on number of copies of GGGTTC. These data were then compared against sequence variants underlying the stature QTL in *B. taurus*: by analyzing crossbred steers descended from two *B. taurus* dairy breeds diverging in height (i.e., small Jerseys and large Holsteins), Karim *et al*.^12^ narrowed the QTL down to eight positional candidate variants (Supplementary File 1). Nishimura *et al*.^17^ also found these variants in Japanese Black cattle, and reported that the direction of the haplotype effect was consistent between studies. These findings implied *Q* to be identical-by-descent across *B. taurus* breeds. An additional implication from the introgression hypothesis was that a single derived haplotype should account for QTL effects in both subspecies of cattle. Here, the coordinates of the associated haplotype region spanned five out of the eight *B. taurus* positional candidates, namely rs110092040 (CHR14:24973953, T > C), ss319607399 (CHR14:24974221, A > G, also rs208989386), ss319607400 (CHR14:24974811, A > G, also rs137303549), rs109231213 (CHR14:25003338, C > G) and ss319607401 (CHR14:25006125, T > C, also rs134215421). The previously reported *B. taurus* haplotype at these variants was *Q* = TAACT. We found that the HD haplotype GGGTTC was in perfect correspondence with the sequence haplotype TAACT in Nellore, suggesting near perfect LD between GGGTTC and *Q* in this breed. Moreover, given that the reference genome animal was most likely *QQ* based on its GGGTTC haplotype, predictions of *qq* genotypes were corroborated by deficit of reference alleles (Fig. 3–c).

### Haplotype diversity in worldwide cattle indicates selection for *Q* in Northwestern Europe

Given the *Q* allele has been recently transferred from *B. taurus* to *B. indicus*, GGGTTC should be the selected haplotype in European breeds and its frequency should be correlated with average body weight and size. In order to test this prediction, we explored haplotype frequencies in the Bovine HapMap data^36,37^, which included HD genotypes from 20 *B. taurus* (n = 503) and two *B. indicus* (n = 65) breeds. Additionally, samples of five *B. taurus* x *B. indicus* crossbred populations (n = 139) and three outgroup species (n = 11) were also available.

The GGGTTC tag was found to be the predominant haplotype in *B. taurus* breeds (Supplementary File 1), consistent with the previously reported selective sweep^9,11^. However, the moderate frequency of GGGTTC in breeds that *a priori* segregate the *Q* mutation at low frequency suggested that LD between GGGTTC and *Q* may be imperfect in *B. taurus* cattle, as opposed to a near perfect LD found in the Nellore population. For instance, Karim *et al*.^12^ estimated that Jersey cattle carry *Q* at a frequency of ~5%, a much lower frequency than 35.9% observed for the GGGTTC haplotype here. Indeed, the authors also reported that the *q* chromosome of one out of four *Qq* sires in their data shared an identical-by-state haplotype with the *Q* chromosome when only SNP array markers were considered. These findings suggest that *Q* frequency predicted from the number of copies of GGGTTC could be overestimated in *B. taurus* as this tag haplotype may also segregate with the ancestral *q* allele at a lower frequency.

In order to address the issue of imperfect LD between GGGTTC and *Q* in *B. taurus*, we took advantage of a variant that is present in the HD array and that is in near perfect LD with the causal mutation in the Jersey and Holstein breeds^12^. This variant, namely rs109815800 (CHR14:25015640, T > G), is positioned immediately after GGGTTC in the HD panel and is located only ~2.9 kbp downstream of the tag haplotype region. Therefore, we attempted to characterize LD between GGGTTC and *Q* by computing the correlation between the tag haplotype and the nucleotide G at rs109815800 in the two mentioned breeds. Our analysis indicated a correlation of *r* = 0.884 (p = 1.02 × 10^−39^), reinforcing the hypothesis that GGGTTC-*Q* occurs at a higher frequency than GGGTTC-*q* in *B. taurus*. Extrapolating from Holstein and Jersey and assuming that the gametic phase of *Q* and G-rs109815800 is at least partially preserved across cattle breeds, we found *r* = 0.889 (*p* = 3.98 × 10^−245^) when all breeds were analyzed simultaneously. We further inspected how often G and T at rs109815800 segregated with GGGTTC in the overall data and found that G-rs109815800 happened almost exclusively with GGGTTC while accounting for 86.9% of all instances of the tag haplotype. Therefore, LD between GGGTTC and *Q* was indeed imperfect but yet substantially high across *B. taurus* populations, which validates this tag haplotype as a proxy for the causal mutation in the present study. However, in order to maximize power, we decided to extend GGGTTC to include G-rs109815800 (GGGTTCG hereafter) for a more robust assessment of the distribution of *Q* in worldwide cattle.

**Figure 4:**
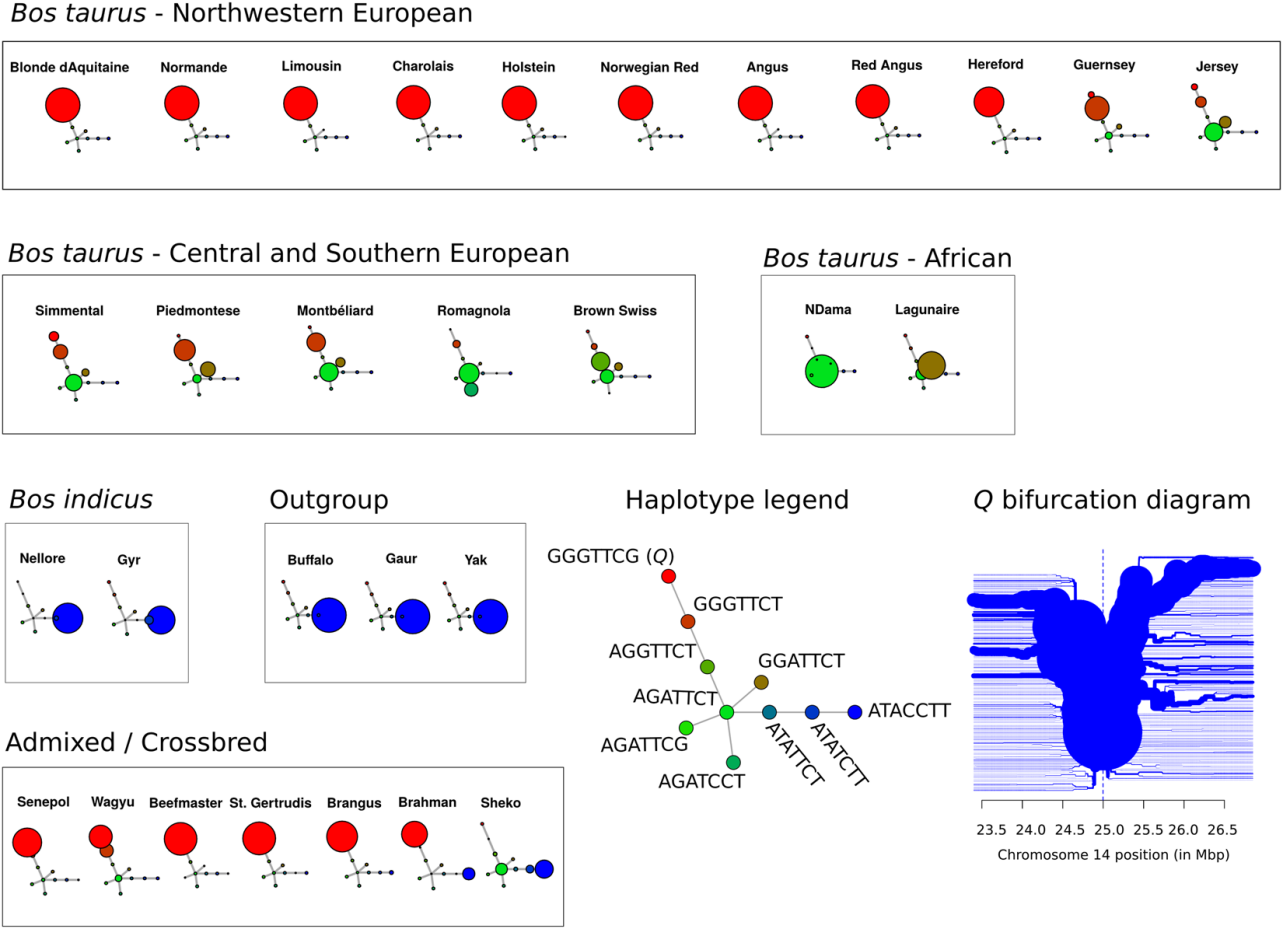
Haplotype diversity at the *PLAG1* locus in the Bovine HapMap data^36,37^. Each node represents a haplotype and edges connect nodes sequentially differing in one or two nucleotides. Node size is proportional to haplotype frequency. The *Q*-tagging haplotype is shown to be highly frequent in breeds originated from Northwestern Europe. A bifurcation diagram (rooted at rs109815800) is also shown, portraying the long-range linkage disequilibrium (LD) and low haplotype diversity around *Q*.

**Figure 5:**
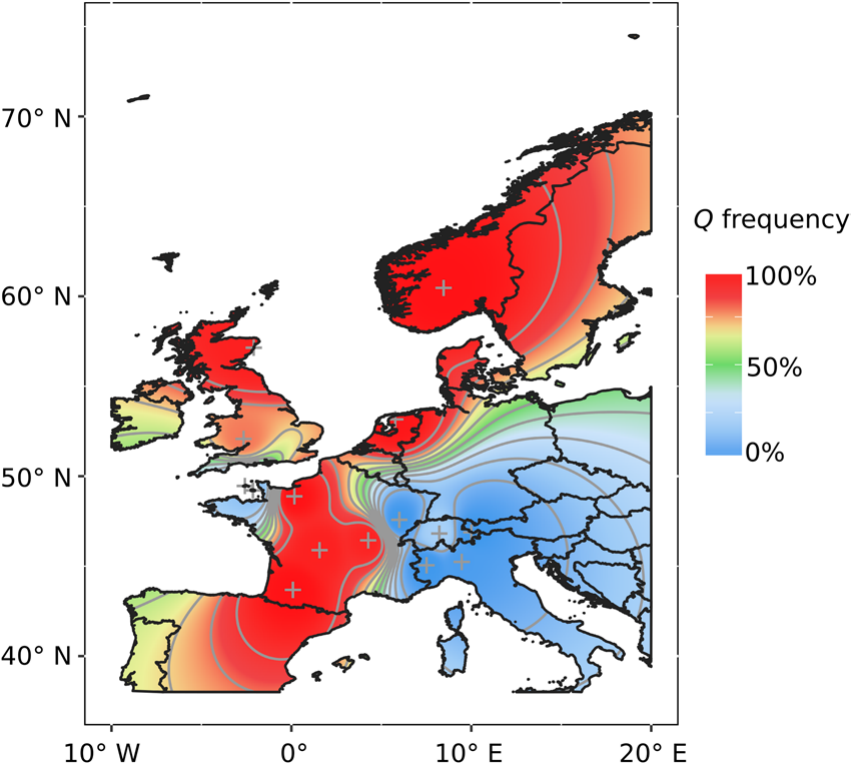
Atlantic Europe as the most likely centre of recent selection for *Q*. The heatmap was generated with ggplot2 v2.1.0^76^ using inverse weighted distance exact interpolation of GGGTTCG frequency from breed origin (crosses).

Frequency of GGGTTCG ranged from 13.0% – 100.0% in Northwestern European cattle (Fig. 4). On the other hand, GGGTTCG was less common in Central and Southern European breeds (0.0% – 20.0%) and even rarer in *B. indicus* (0.0% – 4.0%). Also, GGGTTCG was absent in African *B. taurus*. These data indicated a Northwestern European origin for *Q* or a higher intensity of selection for body weight and size in Northwestern Europe. Haplotype ATACCTT was the most common among *B. indicus* and outgroup populations, confirming its status as ancestral haplotype and supporting further the *B. taurus* origin of *Q*. Additionally, frequency of GGGTTCG in *B. taurus* was positively correlated with average body weight (*r* = 0.607, *p* = 9.99 × 10^−4^) and average withers height (*r* = 0.326, *p* = 0.120), and GGGTTCG was nearly fixed in admixed and crossbred populations selected for body size and weight (Supplementary File 1). Geographical interpolation of the frequency of GGGTTCG further suggested an area facing the Atlantic facade of France, Belgium, Netherlands, Germany, Denmark, Norway, Sweden and Britain as the most likely centre of selection for *Q* (Fig. 5).

### Extended homozygosity and archaeological data indicate a role of *Q* in stature recovery

**Figure 6:**
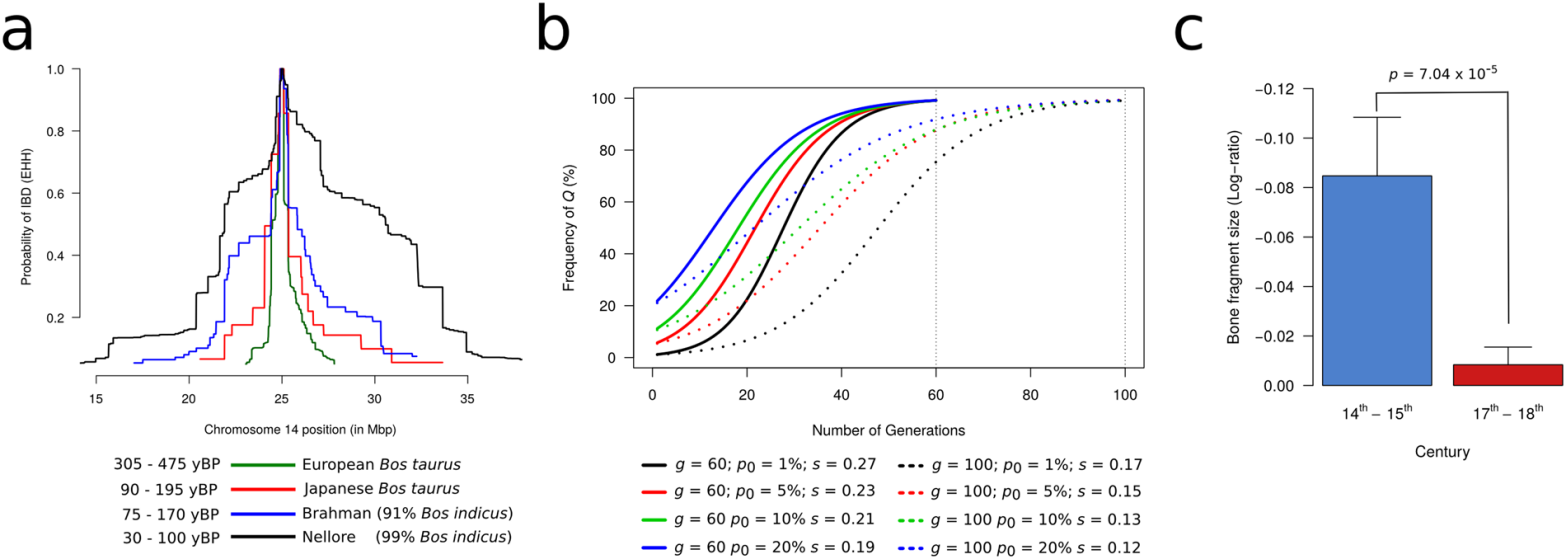
Time to coalescence for the *PLAG1* haplotype. (**a**) Extended haplotype homozygosity (EHH) analysis (rooted at rs109815800). In Northwestern European breeds, the signature dates back to the period of stature recovery in the 16^th^ - 18^th^ century. The haplotype is much more recent in *B. indicus* and Japanese *B. taurus* breeds, indicating introgression. (**b**) Simulation of increase in haplotype frequency according to different selection coefficients (*s*) and frequency prior to selection (*p*_0_). (**c**) Mean log-ratio from bone fragments recovered from archaeological sites in Iceland dating to the 14^th^ – 15^th^ century and the 17^th^ – 18^th^ century. The later period corresponds to early selection for *Q* and presents bone fragments on average 1.2 times larger than those observed in the earlier period. Error bars represent the standard errors of the means.

We used extended haplotype homozygosity^40,41^ to estimate the age of the selective sweep in Northwestern European breeds (Fig. 6a) and found that it dated to ~386 yBP (95% CI [305, 475]). This corresponds to the period between the 16^th^ and 18^th^ centuries, which overlaps with stature recovery in European cattle^1,6^. Simulations suggested that coefficients of selection between 0.12 and 0.27 would be required to rise the frequency of *Q* close to fixation in a time span between 60 (18^th^ century) and 100 (16^th^ century) generations (Fig. 6b). These levels of selective pressure were comparable with those estimated for lactase persistence in humans^42^, indicating strong selection at the *PLAG1* locus. One standing issue, however, is that haplotype coalescence could only provide an estimate of when the haplotype might have started to increase in frequency rapidly, which does not necessarily coincide with the age of the mutation. The identification of carriers of *Q* among animals predating the selective sweep is therefore needed in order to gain insights on the age of the mutation.

Coalescence of the GGGTTCG haplotype in our sample of Nellore bulls was estimated at ~65 yBP (95% CI [30, 100]), consistent with introgression after imports to Brazil from India in the 20^th^ century. Likewise, coalescence in Brahman was ~121 yBP (95% CI [75, 170]), consistent with the period of formation and grading up of this breed. These results provided further evidence of *B. taurus* introgression as a source for *Q* in *B. indicus* populations. We also noticed that coalescence for haplotypes underlying the dominant mutations causing the polled phenotype had intervals matching with those found for *Q* in both Northwestern European and Nellore cattle, which suggested a parallel spread of the *Q* and the polled mutations in worldwide cattle (Supplementary File 1).

The hypothesis of a Northwestern European selective sweep for *Q* could be refuted by the high frequency of GGGTTCG (53.9%) in the Japanese breed Wagyu. Nishimura *et al*.^17^ also reported segregation of the *Q* allele in Japanese Black cattle, and declared an unknown origin of *Q* in Japan. However, Japanese breeds were admixed with Northwestern European cattle between 1868 and 1918^30^. Indeed, analysis of Y chromosome haplotypes in Wagyu revealed multiple admixture events, including introgression from Northwestern cattle (Supplementary File 1). Here, coalescence was younger in Wagyu than in other *B. taurus* breeds and estimated at ~141 yBP (95% CI [90,195]), which falls in the introgression period and supports a Northwestern European origin of *Q* also in Japanese cattle.

If the intense selection for *Q* facilitated stature recovery in Northwestern Europe, a sharp increase in body size should be observed in cattle from the 16^th^ – 18^th^ centuries in comparison to previous centuries. To gain insights on the extent of increase in body size in this period, we analyzed size measurements of bone fragments recovered from two archaeological sites in Iceland^43,44^: (i) Gásir (65° 46′ 58″ N – 18° 9′ 58″ W), containing specimens from the 14^th^ – 15^th^ century (n = 33); and (ii) Skalholt (64° 7′ 38″ N – 20° 31′ 35″ W), containing specimens from the 17^th^ – 18^th^ century (n = 89). In order to make the data comparable across different bone fragments, we computed the log-ratio between measurements taken from each specimen and equivalent measurements from a reference skeleton of a 19^th^ century cow from upstate New York kept at the American Museum of Natural History (specimen #14908, withers height of 94.9 cm). The difference in the mean log-ratio between the 17^th^ – 18^th^ century and 14^th^ – 15^th^ century data was 0.076 ± 0.019 (*p* = 7.04 × 10^−5^), suggesting a ~1.2-fold increase in body size within the time frame when the frequency of *Q* arose rapidly (Fig. 6–c).

### Ancient DNA shows that *Q* has been segregating in *B. taurus* for at least 1,000 years

**Figure 7:**
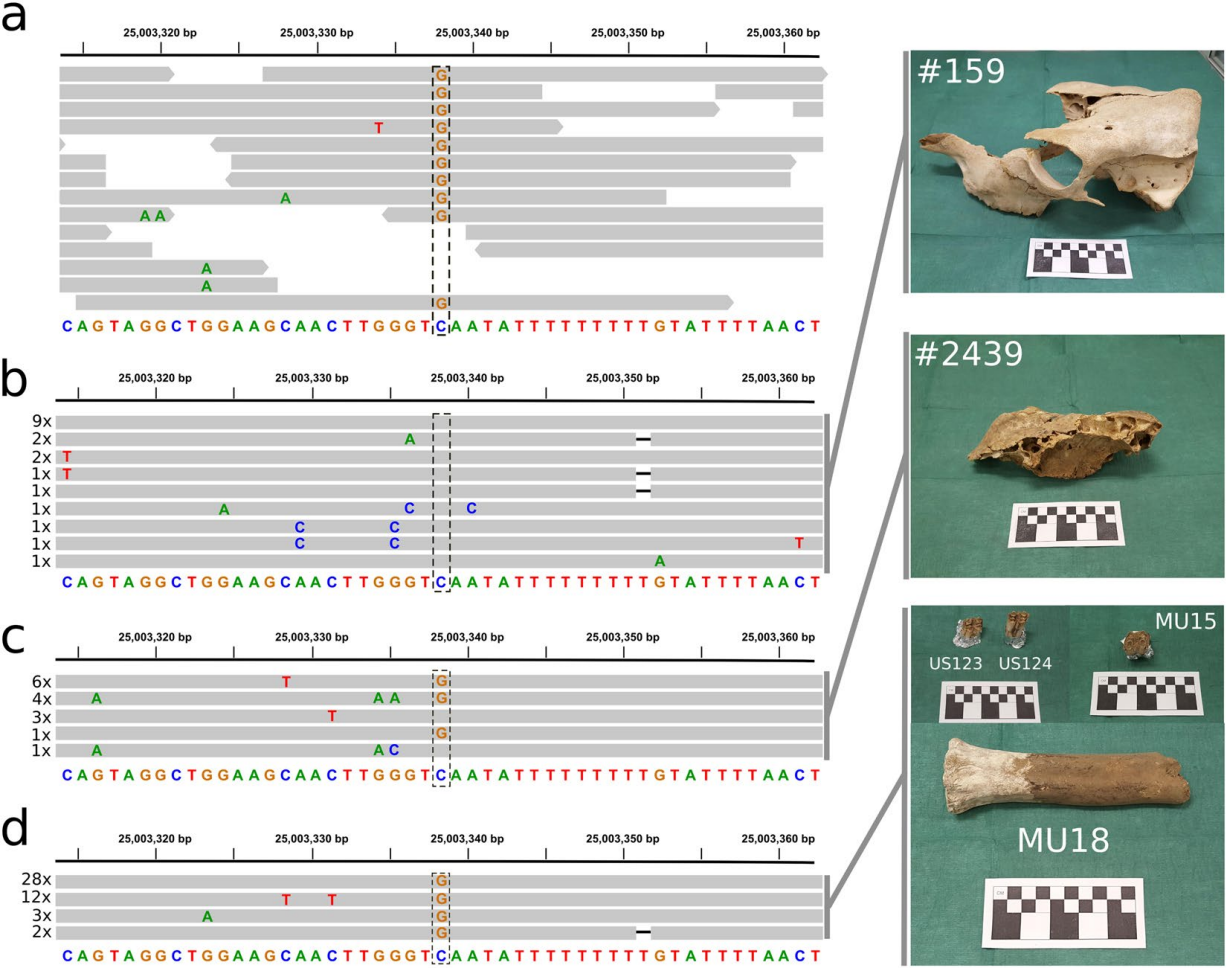
Insights on the age of *Q* from rs109231213 genotypes in ancient DNA. (**a**) Alignments of next-generation sequencing data reported by Park *et al*.^45^ from a >6,000 years old bovine humerus found in England. This specimen carried a *qq* genotype, as evidenced by all reads (horizontal grey bars) presenting allele G at rs109231213 (dashed rectangle). (**b**) A polled cranium (specimen #159) from ~1,000 yBP was recovered from a ritual gathering site in Hofstadir, Iceland^46^. Target-sequencing of its petrous bone revealed a *QQ* genotype (i.e., all clones exhibited allele C at rs109231213). (**c**) A second polled cranium (specimen #2439) recovered from Skalholt and dating >300 yBP returned a heterozygous genotype. (**d**) Two molar teeth from a Medieval site in Northern Italy (US123 and US124) and a molar tooth (MU15) and a long bone (MU18) belonging to the late Roman age (>1,700 yBP) all presented *qq* genotypes.

Insights into the actual age of the *Q* mutation could be gained from the analysis of ancient bovine DNA (aDNA) from Northwestern Europe. In particular, allele C at rs109231213 can be used as a tag for *Q* since this variant affects a highly constrained position (PhastCons score = 1 in 100 vertebrates)^14^ and because it is a functional candidate given its location on the 3′-UTR of *PLAG1* ^12^. We first attempted to find the C allele in short read alignments reported by Park *et al*.^45^ of a well-preserved *Bos primigenius* humerus bone recovered from Carsington Pasture Cave in Derbyshire, England (53° 4′ 47″ N – 1° 38′ 7″ W). This specimen was radiocarbon dated to 6,738 ± 68 yBP and therefore precedes the introduction of domesticated cattle in Britain. Inspection of rs109231213 in their alignment data revealed a clear GG genotype supported by ten single reads, indicating a *qq* genotype (Fig. 7a). In spite of being homozygous wild type, this specimen did not present the ancestral haplotype at HD markers. In fact, the AGATCCT haplotype found in this sample was rare in all modern cattle breeds included in the HapMap set, except for Romagnola. Although Romagnola has been hypothesized to carry *B. indicus* introgression^29,31^, AGATCCT was not found in the *B. indicus* data. This observation reinforced the occurrence of *q* in multiple haplotype backgrounds and indicated that: (i) the specimen was most likely a representative of the non-domesticated Neolithic relative of European *B. taurus* rather than a proxy for *B. primigenius* ancestral to *B. taurus* and *B. indicus*; and (ii) future studies may need to differentiate between introgression from the *B. indicus* lineage and introgression from non-domesticated *B. taurus*. The later is especially important since model-based clustering algorithms tend to cluster *B. indicus* and outgroups together when only two ancestral populations are assumed, in which case hybridization with outgroups becomes indistinguishable from *B. indicus* introgression.

Successful sampling of carriers of *Q* in assemblages of cattle remains predating the selective sweep might be challenging if *Q* was rare prior to selection. However, if our hypothesis of parallel spread of *Q* and polledness holds, DNA extraction from polled specimens may increase the probability of obtaining a CC or CG genotype at rs109231213. Therefore, we extracted aDNA from two naturally polled crania from Iceland. The first specimen dating over 1,000 yBP (#159, Fig. 7b) was recovered from a ritual gathering site in Hofstadir (65° 36′ 47″ N – 17° 10′ 2″ W)^46^, and was considered rare in the sense that polledness was not a common trait for Icelandic cattle from the Viking Age (8^th^ to 11^th^ century). The second specimen (#2439, Fig. 7c) dated over 300 yBP and was collected from the Skalholt site. Sequencing of cloned PCR products including the rs109231213 position revealed the occurrence of allele C in both specimens: the 19 clones obtained from specimen #159 consistently indicated a CC genotype (Fig. 7b), whereas specimen #2439 returned eleven clones with G and four clones with C, indicating a CG genotype (Fig. 7c). Therefore, it seems that both the *Q* and the *POLLED* mutations are at least 1,000 years old.

Since large Roman cattle were introduced in Northwestern Europe during the 1^st^ century, we attempted to detect allele C in aDNA from Northern Italy to test the hypothesis that *Q* was brought from the Central/Southern to the Atlantic region of Europe by the Roman Empire. We analyzed a molar tooth (MU15) and a long bone (MU18) obtained from a late Roman site in Northeastern Italy (46° 7′ 11″ N – 13° 7′ 24″ E) dating over 1,700 yBP. Additionally, two molar teeth (US123 and US124) from different stratigraphic units and thus likely belonging to different individuals from a 13^th^ – 14^th^ century Medieval site in Northern Italy were also analyzed (46° 15′ 54″ N – 10° 26′ 42″ E). All 45 clones obtained from these samples exhibited GG genotypes (Fig. 7d). As our sample size was fairly small, we could not discard occurrence of *Q* in Roman cattle. However, the consistency in the retrieval of GG genotypes indicated that *q* was most likely the major allele in this population.

## Discussion

In the present study we provided evidence that the selective sweep encompassing *PLAG1* dates back to the period of stature recovery in Northwestern European cattle (16^th^ – 18^th^ century)^1^. Also, geographical distribution of *Q* followed a pattern opposite to that of stature loss^7^, reinforcing a role for this mutation in stature recovery. The centre of the selection event for *Q* seems to lie in Atlantic Europe. The actual *Q* mutation is at least one millennium old, as suggested by sequencing of an Icelandic *B. taurus* cranium from ~1,000 yBP.

From its putative centre of origin, our analysis suggested that the *Q* allele spread towards Central, Southern and Eastern Europe, but the intensive selection for pure lines in the past few centuries may have constrained its prevalence in these areas. The *Q* allele was most likely introduced into the New World by colonizers via imports from Northwestern Europe^6^. Cattle trades between Northwestern Europe and Japan in the late 19^th^ and early 20^th^ century^30^ probably favored an introgression of *Q* to Japanese breeds. More recently, *Q* was introgressed into *B. indicus* breeds. For instance, *Q* is speculated to have favored the ‘grading up’ of Brahman cattle, which consisted in introgressing major *B. taurus* alleles into the breed by crossbreeding and back-crossing^14^. Another example provided by our study is Nellore, which gained the *Q* allele after imports to Brazil from crossbreeding with cattle descended from European breeds. This introgression most likely occurred during the population expansion in the late 19^th^ and early 20^th^ century.

**Figure 8:**
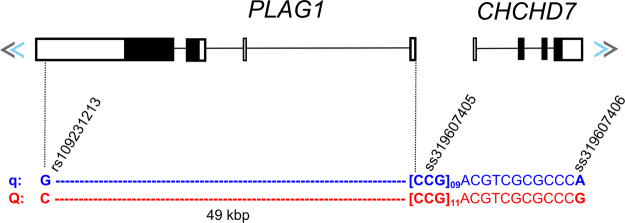
Functional candidate variants underlying the *PLAG1* chromosomal domain. Variant rs109231213 is predicted to change a conserved miRNA binding site, whereas variants ss319607405 and ss319607406 modify the transcription activity of the bi-directional promoter of *PLAG1* and *CHCHD7*.

In spite of our exhaustive analysis, the identity of *Q* remained unknown. Karim *et al*.^12^ reported that none of the eight *B. taurus* positional candidates resided in coding regions or were associated with genotype-specific gene products. However, the authors found haplotype-dependent differences in fetal expression of seven genes, notably *PLAG1*, suggesting a regulon affected by a *cis*-acting causal nucleotide. They further used allelic imbalance and luciferase reporter assays to suggest causality of one of the following three regulatory variants (Fig. 8): (i) rs109231213 (CHR14:25003338), a C to G substitution; (ii) ss319607405 (CHR14:25052396-25052398, also rs209821678), a tandem repeat of eleven or nine CCG copies; and (iii) ss319607406 (CHR14:25052440, also rs210030313), a G to A substitution. All of the three variants changed highly conserved nucleotides across mammals: rs109231213 (PhastCons score = 1) is located at the 3′-UTR of *PLAG1*, whereas ss319607405 (PhastCons score = 0.999) and ss319607406 (PhastCons score = 0.992) are in the bi-directional promoter of coiled-coil-helix-coiled-coil-helix domain containing 7 gene (*CHCHD7*) and *PLAG1*. Still, the individual contributions of these and other variants to the haplotype effect remained unclear.

Interestingly, rs109231213 was the only previously reported functional candidate included in our haplotype region. If not the causal variant itself, our analysis at least suggested that rs109231213 is a reliable proxy. In fact, Fortes *et al*.^14^ have successfully used alleles C and G at rs109231213 as tags for the *Q* and *q* alleles in Brahman, respectively. This is convenient because the other two functional candidates are difficult to type, since they are located in a repetitive GC-rich region. For instance, Nishimura *et al*.^17^ reported difficulties in amplifying the *PLAG1*-*CHCHD7* promoter for sequencing. In the recent selection signatures study by Boitard *et al*.^11^, variants ss319607405 and ss319607406 were not included due to low quality scores and alignment issues. Altogether, these results indicate that in the absence of knowledge about the causal variant, the GGGTTCG haplotype from the HD array or the C allele at SNP rs109231213 could be used to analyze *Q* segregation, trait-association, gene flow, selection and drift in worldwide cattle.

The quest for the identity of *Q* continues. Variants ss319607405 and ss319607406 are appealing candidates since they were shown to change transcriptional activity of the bi-directional promoter of *PLAG1* and *CHCHD7* ^12^. However, rs109231213 is also a strong candidate since it affects a highly conserved nucleotide at the 3′-UTR of *PLAG1*. Variation at the 3′-UTR of a transcript may affect its interaction with regulatory molecules, such as MicroRNAs (miRNA)^47^, and therefore impact levels of translation. Expression levels of *PLAG1* were previously shown to be regulated by miRNAs binding to this 3′-UTR region^48,49^, which also supports candidacy of rs109231213. Indeed, the G to C substitution at rs109231213 is predicted to cause the loss of a highly conserved miRNA binding-site. Another hypothesis involves shared causality of the entire haplotype, meaning *Q* = C(CCG)_11_G and *q* = G(CCG)_9_A, with potential epistatic effects. Separating these effects and solving the causality at this locus will demand the analysis of recombinants at these three loci, which are difficult to find naturally. A recombinant construct between ss319607405 and ss319607406 showed that the (CCG)_11_G haplotype is necessary to change transcription activity^12^, which suggests interaction between the two loci. In order to disentangle interactions between rs109231213 and the other two variants, gene-edited cell lines of G(CCG)_11_G or C(CCG)_9_A recombinants are required and should be the focus of future studies. Moreover, a complex mutation comprising cooperation of multiple regulatory variants at the haplotype region is not yet to be discarded, since other six genes besides *PLAG1* have their expression levels affected to some degree by *Q*. Yet, *PLAG1* seems to be the leading gene behind most of the pleiotropic effects observed at this chromosomal domain, as suggested by the recent expression QTL study of Fink *et al*.^50^. Finally, previously unavailable functional annotations such as expression QTLs, promoters, enhancers, insulators, transcription factor binding sites, CTCF loops and 3D chromatin structure, among others, will be soon available from the upcoming *B. indicus* assembly, the new long-read *B. taurus* assembly and the Functional Annotation of Animal Genomes (FAANG) projects. As our understanding of regulatory elements in the bovine genome progresses, other candidate variants may also emerge and reveal additional trait-specific effects.

In conclusion, we were able to demonstrate that the pleiotropic QTL and signature of selection spanning *PLAG1* are most likely explained by a single mutation (or haplotype) across modern worldwide cattle breeds. We also found that this mutation has been selected during the period of stature recovery in Northwestern Europe and was later spread around the globe via importation of cattle from Atlantic Europe. Altogether, this study presents one of the first examples of a selective sweep in livestock that was driven by strong (supposedly artificial) selection on a complex trait.

## Methods

### Phasing and haplotyping

Nellore data were filtered in PLINK v1.90^51,52^ for a minium call rate of 95% and minor allele frequency of at least 5%. A total of 447,617 markers (out of 786,799) were retained for analysis. Genotypes were phased with the Segmented HAPlotype Estimation & Imputation Tool v2.r837 (SHAPEIT2)^33^. The following parameter values were used: effective population size (Ne_LD_) of 113, burn in of ten iterations, prune of ten iterations, 50 main iterations, 200 states, and windows of 500 kbp. Effective population size was estimated from the data with SNeP v1.1^53^. Phasing with default parameters and a previously published value for Ne_LD_ of 362^54^ based on a larger sample of Nellore animals yielded very similar results (data not shown). We used GHap v1.2.2^55^ to determine haplotype alleles within chromosomal segments and score haplotype genotypes for each animal. The segment size was chosen based on the extent of LD in the Nellore genome and the intermarker spacing of the HD panel. Given that markers were placed on average every ~5 kbp on CHR14, and that LD extends up to 30 kbp in the Nellore genome^56^, we determined haplotypes within overlapping segments of six consecutive markers. A total of 15,127 sliding windows of six consecutive markers were screened throughout autosome 14 for short haplotype calling. The same phasing and short haplotype calling procedures were later applied to CHR1 and HapMap genotypes, with Ne_LD_ reset to the default value of 10,000 in the later case.

### Phenotypes

Birth weight estimated breeding values were obtained from a single-trait animal model fitted to records from 846,782 calves born between 1985 and 2012 in 315 grazing-based Brazilian herds. The model included the fixed effects of contemporary group (defined as animals from the same herd, born in the same year and season, and belonging to the same birth management group) and age of dam at calving, as well as random maternal effects (maternal additive genetic effect and maternal permanent environmental effect)^18^. The heritability was estimated at 0.37. Prior to the association analysis, estimated breeding values were deregressed following Garrick *et al*.^57^. The minimum, mean, standard deviation and maximum of the accuracy of dEBV (based on prediction error variance) was 0.58, 0.95, 0.30 and 0.98, respectively. Presence or absence of horns was scored by five independent evaluators from pictures of the genotyped Nellore bulls. Majority voting was used to assign phenotypes to animals, and at least three voters were required to declare an animal to be horned or polled. Furthermore, pictures from animals suspected to be surgically de-horned were excluded. The resulting data included 328 horned and 51 polled bulls.

### Haplotype regression analysis

The parameterization presented here builds on previously reported models^58,59^. For a given window, let haplotypes 1, 2, …, *K* be sorted by frequencies *p*_1_, *p*_2_, …, *p*_K_. We start by defining α_k_ as the average effect of substituting the minor haplotype 1 by *k* = 2, 3, …, *K*. From these definitions, the partial breeding value associated with haplotype *k* is:

1 $${u}_{{\rm{k}}}={z}_{{\rm{k}}}{a}_{{\rm{k}}}$$

where *z*_k_ is a scalar taking values:

0 – 2*p*_k_, for 0 copies of haplotype *k*

1 – 2*p*_k_, for 1 copy of haplotype *k*

2 – 2*p*_k_, for 2 copies of haplotype *k*

phenotypes were regressed onto haplotype-specific breeding values using the model:

2 $${\bf{y}}={\bf{X}}{\bf{b}}+{\bf{e}}$$

where **y** is the vector of phenotypes, **b** = [μ α_k_]′, μ is an intercept, **X** = [**1 z**_k_], **1** is a vector of ones, **z**_k_ is a vector relating phenotypes to substitution effect α_k_, and **e** is a vector of residuals distributed as *N*(0,**W**σ^2^_e_), where **W** = Diag(**w**), **w** is a vector of weights, and σ^2^_e_ is the residual variance. In the case of birth weight dEBVs, the vector of weights was defined as **w** = λ^−1^(**d** + *c*)^57^, where λ = (1 − *h* ^2^)/*h* ^2^, *h* ^2^ is the heritability of birth weight, **d** = (1 − **r** ^2^)/**r** ^2^, **r** ^2^ is the vector of dEBV reliabilities, and *c* is the assumed proportion of genetic variance for which haplotypes cannot account. We have previously estimated^18^ a 95% confidence interval of 2.12-8.09% for the genetic variance due to the targeted QTL. Therefore, we adopted a value of *c* = 0.9191 (i.e., the candidate haplotype can only explain at most 8.09% of the genetic variance). For the polledness analysis, horned and polled animals had phenotypes coded as −1 and 1, respectively, and **W** was replaced by an identity matrix. Regression was carried out by solving the generalized least squares equations **b** = (**X**′**W** ^−1^ **X**)^−1^ **X**′**W** ^−1^ **y**. Association mapping was then based on the two-tailed t-test α_k_ / SE(α_k_), and the most significant haplotype was selected as a tag for the underlying causal mutation. Similar results were found when association tests were performed using haplotype windows of 1 (i.e., single SNP), 5, 10 and 20 markers with correction for polygenic effects (Supplementary File 1).

### Analysis of ancestry and genetic structure

The unsupervised model-based clustering algorithm in Admixture 1.3^35^ was used to estimate *B. taurus* ancestry on CHR14 in Nellore cattle. The HapMap data was merged with the Nellore data and the algorithm was run assuming two ancestral populations (*B. taurus* and *B. indicus*). Admixed and outgroup populations were excluded from this analysis. Principal components were computed with PLINK v1.90^51,52^. Statistical tests for differences in *B. taurus* ancestry on CHR14 and mean birth weight dEBV between breeding subgroups of Nellore cattle were based on a Mann-Whitney U test and a t-test, respectively. Differences in frequency of *Q* and polledness were assessed with Fisher’s exact test. Local ancestry was estimated using the same reference data with elai v1.00^60^. Ten independent runs with different random seeds were performed in parallel, from which eight were based on the Expectation-Maximization (EM) algorithm and two were obtained using a fast linear approximation. The EM algorithm was run with 30 steps. All analyses assumed ten generations since admixture and numbers of upper and lower clusters of two and ten, respectively. The final output was built from the average across the ten replicates.

### Extended haplotype homozygosity

For each marker pertaining to the haplotype of interest, and considering only chromosomes carrying that haplotype, the decay in the probability of identity-by-descent was modeled using extended haplotype homozygosity (EHH), following Sabeti *et al*.^41^. Briefly, EHH between a core marker and another marker upstream or downstream was computed as:

3 $$EHH=\frac{\sum _{h=1}^{H}(\begin{array}{c}{n}_{h}\\ 2\end{array})}{(\begin{array}{c}N\\ 2\end{array})}$$

where (:) denotes the binomial coefficient, *n*_h_ is the count of haplotype *h* carrying the allele of interest and *N* is the total number of chromosomes carrying that allele. Calculation of EHH was repeated for all markers upstream and downstream of a core marker until EHH decayed to 0.05, corresponding to a 5% probability of obtaining a pair of haplotypes identical by descent when two chromosomes were sampled at random. All EHH calculations were performed using the *rehh* v2.0.0 R package^61^. The expected number of generations until coalescence was obtained as^40^:

4 $${\rm{E}}[g]=1-\,\mathrm{ln}(p)/r$$

where *p* is the probability of identity-by-descent and *r* is the segment size in Morgans. The latter was computed from the entire length span of the EHH decay assuming 1 cM ~ 1.23 Mbp^62^. The use of a genetic map to account for local recombination rates yielded very similar results (Supplementary File 1). Time to haplotype coalescence was computed based on a cattle generation interval of five years. Confidence intervals were derived from 1,000,000 Monte Carlo simulations assuming that the number of generations followed a Poisson distribution with parameter λ = E[*g*]. These simulations were verified to be equivalent to assuming estimate error *e* ~ *N*(0, λ).

### Coefficient of selection

The magnitude of selection required to fix the *Q* mutation in the course of 60 or 100 generations was computed considering the equation relating average fitness in a population (*w*) to allele frequencies under constant selection^40^:

5 $$w={p}^{2}{w}_{QQ}+2p(1\,-\,p){w}_{Qq}+{(1-p)}^{2}{w}_{qq}$$

where *p* is the frequency of *Q*, *w*_*QQ*_ = 1, *w*_*Qq*_ = 1 − *hs* and *w*_*qq*_ = 1 – *s* are the relative fitness of genotypes *QQ*, *Qq* and *qq*, respectively, *h* is a scalar taking values 0, 0.5 or 1 if the effect of *Q* was assumed dominant, additive or recessive, respectively, and *s* is the coefficient of selection. Based on the additive distribution of phenotypes according to genotypes, we adopted *h* = 0.5. Change in *p* from one generation to the next was then computed as:

6 $${p}_{{\rm{t}}+1}={p}_{{\rm{t}}}[{p}_{{\rm{t}}}{w}_{QQ}+(1\,-\,{p}_{{\rm{t}}}){w}_{Qq}]/w$$

We simulated a range of scenarios with different frequencies for *Q* at *t* = 0, starting at 1% and going up to 20%. For each scenario, values of *s* ranging from 0.01 to 0.50 were tested in order to identify a coefficient of selection sufficient to increase the frequency of *Q* up to 99% in 60 or 100 iterations.

### Analysis of bone measurements

The archaeological data used for size analysis comprised fragments of humerus (n = 38), tibia (n = 27), metatarsal (n = 24), metacarpal (n = 11), calcaneus (n = 2), femur (n = 15) and radius-ulna (n = 5) bones. According to bone type and integrity, measurements included breadth of the distal end (Bd, n = 14), breadth of the proximal end (Bp, n = 13), breadth of trochlea (BT, n = 13), depth across the processus anconaeus (DPA, n = 10), greatest breadth (GB, n = 15), greatest length (GL, n = 18), smallest breadth of diaphysis (SD, n = 16) and smallest depth of olecranon (SDO, n = 12). Equivalent measurements were taken from a 19^th^ century reference cow skeleton (specimen #14908 from the American Museum of Natural History) and the ratio between specimen and reference was calculated. Ratios were then transformed to a log10 scale. A t-test was used to compare the log-ratio means between specimens from the 17^th^ – 18^th^ century and the 14^th^ – 15^th^ century.

### Analysis of ancient DNA

A total of seven samples were used for aDNA analysis: two petrous bones from a 10^th^ century bovine cranium (specimen #159) from the site of Hofstadir and one petrous bone from a 15^th^–17^th^ century bovine cranium (specimen #2439) from the site of Skalholt; one molar tooth (MU15) and one long bone (MU18) from the 3^rd^ – 4^th^ century from the late Roman site of Muris di Moruzzo (UD) in Northeastern Italy (46° 7′ 11″ N – 13° 7′ 24″ E); and two molar teeth (US123 and US124) from different stratigraphic units and thus likely belonging to different individuals from the 13^th^ – 14^th^ century Medieval site of Tor dei Pagà (Vione, BS) in Northern Italy (46° 15′ 54″ N – 10° 26′ 42″ E). Extraction, PCR amplification and cloning of aDNA were performed in a dedicated lab facility of the BioDNA Research Centre (Piacenza, Italy). To exclude contamination from exogenous sources, stringent criteria for aDNA analysis were followed: extraction and PCR set up were carried out in physically separated clean rooms, irradiated with UV light (254 nm wavelength) each night for 2 h and additionally after every work session, and equipped with a positive air pressure system to prevent external contaminants from entering. All post-PCR analyses were performed in a separate modern-DNA lab. Disposable Tyvek® coveralls and double gloves were worn during the experiments and were changed frequently. All benches and rooms were routinely treated with bleach and UV-light. Extraction and amplification blanks were used as negative controls in each experiment. In the “extraction” clean room, after a 2 h UV light preliminary irradiation of the samples, the external layer of the surface was removed with disposable burs on a Marathon Multi 600 dental device. The samples were further UV-irradiated for 45 minutes and ground into powder with the same tool. Starting from about 250 mg of teeth/bone powder per sample, DNA was extracted according to the method of Rohland and Hofreiter^63^. Amplification reactions were assembled in the “PCR set up” clean room under a Biosan UVC/T-AR cabinet. PCR amplification of a 104 bp fragment flanking the target polymorphism (rs109231213) was obtained with the primer pair Bt_PLAG1_F 5′ CTCAAAACACACTGTCTTCCCA 3′ and Bt_PLAG1_R 5′ GATCTCCTCCAATGTCGCCT 3′. Two ml of extracted DNA were added to 50 ml PCR reaction mix including 2U of AmpliTaq Gold® DNA Polymerase (Applied Biosystems), 2 mM MgCl_2_, 160 μM of each dNTP and 1 μM of each primer. The amplification thermal profile was as follows: 95 °C for 10 min (polymerase activation), 45 cycles of denaturation, 95 °C for 20 sec, annealing, 50 °C for 20 sec and extension, 72 °C for 30 sec, and final extension at 72 °C for 10 min. PCR products were checked on 1.5% agarose gel. Bands of expected size were excised from agarose gel, purified with Sepharose® Cl-6B resin (GE Healthcare) on Micro Biospin Columns 732-6204 (BIORAD) and cloned with the TOPO® TA Cloning® Kit (Invitrogen^TM^) following the manufacturer’s instructions. A total of 10 to 15 clones per sample were amplified from white recombinant colonies with universal M13 primers. After purification with Sepharose® resin, PCR products were sequenced with M13 forward primer in outsourcing at Macrogen company (http://www.macrogen.com). The resulting chromatograms were visually inspected with SeqTrace v0.9.0^64^ and aligned to the *PLAG1* sequence of the cattle reference genome with BioEdit v7.2.5^65^.

### Analysis of next-generation sequencing data

Paired-end libraries of 24 Nellore bulls were sequenced in the Illumina® HiSeq. 2000 platform, following the manufacturer’s protocol^66^. Reads were aligned against the UMD v3.1 *B. taurus* assembly^10^ using the Burrows-Wheeler Alignment (BWA) algorithm v0.7.10-r789^67^. After alignment, optical and PCR duplicates were marked with PicardTools v1.119^68^. The total sequencing yield was 2 × 3,205,981,178 paired-end reads of 100 bases (~641 billion bases). Considering only properly paired reads, 92.2% were successfully mapped, resulting in an average fold coverage per sample of 9.25 ± 1.48x, with a minimum of 5.87x and a maximum of 13.75x. The overall percentage of optical/PCR duplicates was 7.0%. Variants on CHR14 were extracted from aligned reads using the mpileup algorithm from SAMtools v1.3.1 and BCFtools v1.3.1^69^, following guidelines for cattle data reported by Baes *et al*.^70^. Variant effects were predicted and annotated with Ensembl Variant Effect Predictor (VEP)^71^. Sequence alignments were visually inspected to confirm variant positions with both Integrative Genomics Viewer (IGV) v2.3 tool^72,73^ and the SAMtools v1.3.1 tview application^69^.

### Data availability

The data that support the findings of this study were obtained under license and so are not publicly available. Data are however available for academic use from the authors upon reasonable request.

## Electronic supplementary material

Supplementary File 1
