# Supplementary material for: A PLAG1 mutation contributed to stature recovery in modern cattle
Source: Sci Rep. 2017 Dec 7;7:17140. doi: 10.1038/s41598-017-17127-1 (PMC5719367; doi:10.1038/s41598-017-17127-1)
Supplement: Supplementary file 1 [file 41598_2017_17127_MOESM1_ESM.pdf]

Supplementary file 1

A *PLAG1* mutation contributed to stature recovery in modern cattle

Yuri Tani Utsunomiya[1,2]*, Marco Milanesi[2,3,4], Adam Taiti Harth Utsunomiya[2,4], Rafaela Beatriz Pintor Torrecilha[1,2], Eui-Soo Kim[5], Márcio Silva Costa[6], Tamíris Sayuri Aguiar[1,2], Steven Schroeder[7], Adriana Santana do Carmo[8], Roberto Carvalheiro[9], Haroldo Henrique de Rezende Neves[10], Romulo Cláudio Morozini Padula[11], Thayla de Souza Sussai[11], Ludmilla Balbo Zavarez[1,2], Rafael Silva Cipriano[11], Maria Margareth Theodoro Caminhas[4], George Hambrecht[12], Licia Colli[3], Elisa Eufemi[3], Paolo Ajmone-Marsan[3], Deneb Cesana[13], Marco Sannazaro[13], Maurizio Buora[14], Michele Morgante[15,16], George Liu[7], Derek Bickhart[7], Curtis Paul Van Tassell[7], Johann Sölkner[17], Tad Stewart Sonstegard[5], José Fernando Garcia[1,2,4]*

[1]São Paulo State University (Unesp). School of Agricultural and Veterinarian Sciences, Jaboticabal. Department of Preventive Veterinary Medicine and Animal Reproduction. São Paulo, Brazil;
[2]International Atomic Energy Agency (IAEA) Collaborating Centre on Animal Genomics and Bioinformatics, Araçatuba, São Paulo, Brazil;
[3]Istituto di Zootecnica and BioDNA Centro di Ricerca sulla Biodiversità e sul DNA Antico, Università Cattolica del Sacro Cuore, Piacenza, Italy
[4]São Paulo State University (Unesp). School of Veterinary Medicine, Araçatuba. Department of Support, Production and Animal Health. São Paulo, Brazil;
[5]Recombinetics, Inc., St Paul, MN 55104, USA;
[6]UFPI – Universidade Federal do Piauí, Piauí, Brazil;
[7]Animal Genomics and Improvement Laboratory, Agricultural Research Service, USDA, Beltsville, Maryland, USA;
[8]Escola de Veterinária e Zootecnia (EVZ), Universidade Federal de Goiás - UFG, Campus Samambaia, Goiânia, Goiás, Brazil;
[9]São Paulo State University (Unesp). School of Agricultural and Veterinarian Sciences, Jaboticabal. Department of Animal Science. São Paulo, Brazil;
[10]GenSys Consultores Associados, Porto Alegre, Brazil;
[11]Centro Universitário Católico Salesiano, Araçatuba, São Paulo, Brazil;
[12]Department of Anthropology, University of Maryland, USA;
[13]Dipartimento di Storia, Archeologia e Storia dell'Arte, Facoltà di Lettere e Filosofia, Università Cattolica del Sacro Cuore, Milano, Italy;
[14]Società Friulana di Archeologia, Udine, Italy;
[15]Istituto di Genomica Applicata, Udine, Italy;
[16]Dipartamento di Scienze Agrarie ed Ambientali, Università di Udine, Udine, Italy;
[17]BOKU - University of Natural Resources and Life Sciences, Department of Sustainable Agricultural Systems, Division of Livestock Sciences, Vienna, Austria;

*Correspondence should be addressed to Y.T.U. (ytutsunomiya@gmail.com) or J.F.G. (jfgarcia@fmva.unesp.br)

1. Analysis of Y chromosome haplotypes supports introgression of *Q* in Japanese cattle

The HapMap data are summarized in **Supplementary Table 1**. The Illumina® BovineHD panel included 1,224 Y-linked markers. Ideally, genotypes at these markers should be recalled manually since both the standard cluster file and the GenTrain2 algorithm in GenomeStudio assume diploidy. However, as this process is extremely laborious, we opted to analyze only markers with obvious two-states hemizygous genotypes in the HapMap data. First, we retained only markers with GenTrain Score greater than 0.80. This threshold guaranteed that clusters AA, AB and BB were well-shaped and clearly separated. Second, animals with call rate lower than 95% at Y-linked markers were excluded, which mainly caused females to be removed from downstream analyses. Next, all remaining heterozygous calls were set to missing and homozygous genotypes were replaced by hemizygous states (0 for AA and 1 for BB). Lastly, only markers with call rate equal to 100% and minor allele frequency greater than 5% in the overall breed data were preserved. This filtering procedure resulted in 29 markers and 563 individuals, which yielded five different haplotypes (YHapG1 – YHapG5, **Supplementary Table 2**).

YHapG1 and YHapG2 were identified as *B. indicus* haplotypes, whereas YHapG3, YHapG4 and YHapG5 were recognized as *B. taurus* haplotypes. Additionally, YHapG3 was nearly fixed in six out of the nine Northwestern European breeds that were highly selected for *Q*. Importantly, similar to crossbred and admixed populations, Wagyu presented multiple Y chromosome haplotypes, including YHapG3, which is consistent with admixture and supports the hypothesis of introgression of *Q* from Northwestern European cattle into Japanese breeds.

Supplementary Table 1 Breed information for the Bovine HapMap data

Species	Breed	Origin	Longitude[a]	Latitude[a]	Purpose	Sample size	Weight (in kg)[b]	Withers height (in cm)[b]
Bos taurus	Blonde d'Aquitaine	France	0.0878906	43.6821749	Beef	5	1300	165
	Normande	France	0.1712529	48.8798704	Dairy	5	1100	155
	Norwegian Red	Norway	8.4689460	60.4720240	Dairy	17	1000	142
	Red Angus	Britain	-2.0942780	57.1497170	Beef	11	1000	145
	Holstein	Netherlands	5.7817542	53.1641642	Dairy	71	1100	165
	Angus	Britain	-2.0942780	57.1497170	Beef	47	1000	145
	Limousin	France	1.5696018	45.8932231	Beef	51	1050	144
	Charolais	France	4.2752780	46.4344400	Beef	40	1150	146
	Senepol	Caribbean (composite)	-64.8347992	17.7245968	Beef	12	930	136
	Wagyu	Japan	138.2529240	36.2048240	Beef	13	940	142
	Hereford	Britain	-2.6544182	52.0765164	Beef	38	1050	135
	Guernsey	Britain	-2.5852780	49.4656910	Dairy	21	760	140
	Simmental (Fleckvieh)	Switzerland	8.2275120	46.8181880	Dairy	10	1200	152
	Piedmontese	Italy	7.5153885	45.0522366	Beef	24	950	150
	Montbéliard	France	6.0000000	47.5833300	Dairy	5	1100	148
	Jersey	Jersey	-2.1312500	49.2144390	Dairy	46	600	127
	Romagnola	Italy	9.4702148	45.2377644	Beef	34	700	145
	Brown Swiss (Braunvieh)	Switzerland	10.0000000	46.8812215	Dairy	24	1050	152
	N'Dama	Guinea	-9.6966450	9.9455870	Dairy	24	370	116
	Lagunaire	Benin	2.3158340	9.3076900	Beef	5	200	100
Bos indicus	Nellore	India	79.9864560	14.4425987	Beef	35	570	150
	Gyr	India	71.1923805	22.2586520	Dairy	30	544	140
Bos taurus x Bos indicus	Beefmaster	United States (composite)	-99.9018131	31.9685988	Beef	24	1202	NA
	Santa Gertrudis	United States (composite)	-97.8561090	27.5158689	Beef	35	861	NA
	Brahman	United States (composite)	-81.1637245	33.8360810	Beef	49	887	126
	Brangus	United States (composite)	-91.6634483	29.9110378	Beef	13	900	135
	Sheko	Ethiopia (ancient crossbred)	40.4896730	9.1450000	Dairy	18	NA	NA
Bubalus bubalis	Buffalo	Southern Asia	71.1923805	22.2586520	Dual	7	NA	NA
Bos gaurus	Gaur	Southern Asia	76.4563087	25.0376400	Dual	2	NA	NA
Bos grunniens	Yak	Southern Asia	91.1175250	29.6475350	Dual	2	NA	NA

[a]Longitude and latitude data were approximated based on the geographical origin of each breed.
[b]Average male body weight and withers height data were obtained from the Domestic Animal Diversity Information System of the Food and Agricultural Organization (FAO) of the United Nations. Available at: http://dad.fao.org/. Accession date: 10 Nov 2016.

Supplementary Table 2 Frequency of Y chromosome haplotypes[a] in the HapMap data

Breed	YHapG1: GTCTCCCGCCGGATAG GGCGCAGCCCACT	YHapG2: GTCTCCTACCGGATAG GGCGCACCCCACT	YHapG3: ACTCGGTATTACGCAA AATATGCTTCTAC	YHapG4: ACTCGGTATTACGCTA GATATGCTTCAAC	YHapG5: ACTCGGTATTACGCAA GATATGCTTTAAC
Blonde d'Aquitaine	0.0000	0.0000	0.0000	1.0000	0.0000
Normande	0.0000	0.0000	1.0000	0.0000	0.0000
Norwegian Red	0.0000	0.0000	1.0000	0.0000	0.0000
Red Angus	0.0000	0.0000	1.0000	0.0000	0.0000
Holstein	0.0000	0.0000	1.0000	0.0000	0.0000
Angus	0.0000	0.0000	1.0000	0.0000	0.0000
Limousin	0.0000	0.0000	0.0000	1.0000	0.0000
Charolais	0.0000	0.0000	0.0000	1.0000	0.0000
Senepol	0.0000	0.0000	0.1667	0.8333	0.0000
Wagyu	0.0000	0.0000	0.1538	0.5385	0.3077
Hereford	0.0000	0.0000	0.7576	0.0606	0.1818
Guernsey	0.0000	0.0000	0.0000	1.0000	0.0000
Simmental (Fleckvieh)	0.0000	0.0000	0.2000	0.8000	0.0000
Piedmontese	0.0000	0.0000	0.0000	0.6250	0.3750
Montbéliard	0.0000	0.0000	0.0000	1.0000	0.0000
Jersey	0.0000	0.0000	0.1395	0.0000	0.8605
Romagnola	0.0000	0.0000	0.0000	1.0000	0.0000
Brown Swiss (Braunvieh)	0.0000	0.0000	0.0000	1.0000	0.0000
N'Dama	0.0000	0.0000	0.0000	1.0000	0.0000
Nellore	1.0000	0.0000	0.0000	0.0000	0.0000
Gyr	1.0000	0.0000	0.0000	0.0000	0.0000
Beefmaster	0.3043	0.0000	0.6957	0.0000	0.0000
Santa Gertrudis	0.9688	0.0000	0.0000	0.0313	0.0000
Brahman	1.0000	0.0000	0.0000	0.0000	0.0000
Brangus	0.0000	0.0000	0.8571	0.1429	0.0000
Sheko	0.6000	0.4000	0.0000	0.0000	0.0000

[a]Haplotypes were defined based on a subset of 29 polymorphic markers mapping to chromosome Y.

2. Coalescence at the *POLLED* locus implies parallel selection for *Q* and polledness

Two candidate mutations causing polledness in *B. taurus* have been previously identified on chromosome 1 (CHR1) [1,2]: an ~80 kbp duplication of Friesian origin (P_F, CHR1:1909352-1989480) and an 212 bp duplication of Celtic origin (P_C, CHR1:1705834-1706045). Similarly to *Q*, both mutations appear to occur in a Northwestern European selective sweep spanning CHR1:1693164-2018403. Also, the earliest predicted carrier of *Q* in our Nellore data was polled, raising the hypothesis of concomitant spread of polledness and *Q* in worldwide cattle. The main caveat in the analysis of the *POLLED* locus is that both short-horned and polled breeds present loss of diversity with similar haplotypes in this CHR1 region [2], indicating that a putative selection event for decreased horn size preceded the occurrence of both P_C or P_F and that other variants in the region might also contribute to horn size and morphology. In this case, identical-by-state haplotypes constructed from array data can be in linkage with P_C, P_F or even with the short-horn allele, preventing reliable estimation of identity-by-descent. Nevertheless, it was still possible to use haplotype analyses to estimate coalescence of the selection signatures for decreased horn size and polledness in European cattle and detect *B. taurus* introgression at this locus in the *B. indicus* lineage.

First, in order to confirm whether the polled allele in Nellore was indeed of *B. taurus* origin, we regressed horned/polled phenotypes onto CHR1 haplotypes. Prior to phasing, misassembled segments on CHR1 were excluded, following our previous report [3]. Associations mapped to a ~559 kbp segment on CHR1:1473797-2032837 (p = 2.28 x 10^{-28}), which included the relevant signature region (**Supplementary Fig. 1a**). To simplify the analysis, we focused on the six-markers haplotypes overlapping with the positions of P_C (CHR1:1702350-1721777) and P_F (CHR1:1905048-1925652). At the P_C and P_F regions, the significant haplotypes were TGAAAG and TGTAGG, respectively. Both were the major haplotypes in most of the European breeds in the HapMap data, except for Romagnola (see **Supplementary Tables 3** and **4**). These haplotypes were also rare or absent in African *B. taurus*, *B. indicus* and outgroup species. This confirms that polledness in Nellore is a *B. taurus* contribution.

Frequency of the *PLAG1* haplotype GGGTTC was highly correlated with frequency of TGAAAG or TGTAGG (r = 0.787, p = 2.53 x 10^{-7}) in worldwide cattle (**Supplementary Table 5**). Although the frequencies of GGGTTC (0.1778) and TGAAAG/TGTAGG (0.1007) were similar in our sample of Nellore bulls, GGGTTC occurrence did not differ between carriers (0.224) and non-carriers (0.181) of TGAAAG/TGTAGG. Since *POLLED* and *PLAG1* were located in different chromosomes, independent assortment of these haplotypes may have favored an even spread of *Q* between polled and horned Nellore after introgression. This is plausible given selection for polledness is currently practiced by specific groups of breeders.

Considering all Northwestern European breeds together (**Supplementary Fig. 1b**), time to coalescence of the two *POLLED* haplotypes was estimated at ~2274 yBP (95% CI [2070, 2485]), which implies selection for decreased horn size since the Iron Ages. However, when only polled breeds carrying the Celtic allele were considered (i.e., Angus, Red Angus and Simmental), coalescence was much younger, indicating a selective sweep approximately ~464 yBP (95% CI [370, 560]). Considering the Friesian allele (i.e., Holstein and Jersey), the signature dated to ~565 yBP (95% CI [445, 650]). These estimates were close to the coalescence for the *Q* haplotype. Although we still cannot directly connect the origin of the *Q* mutation to the origin of P_C/P_F, altogether our results provide evidence that increased body size and polledness were selected in parallel in Europe. This implies that polled European cattle are likely to carry *Q*, and that either early carriers were polled or that polledness first appeared in populations selected for *Q*.

The estimate in Nellore carriers was ~203 yBP (95% CI [145, 270]), suggesting that introgression of TGAAAG/TGTAGG occurred earlier than *Q*, most likely as soon as the first imported animals arrived from India to Brazil. Of note, due to the cryptic allelic heterogeneity at the *POLLED* haplotypes, we could not link this time estimate with the actual introgression of the polled phenotype into Nellore.

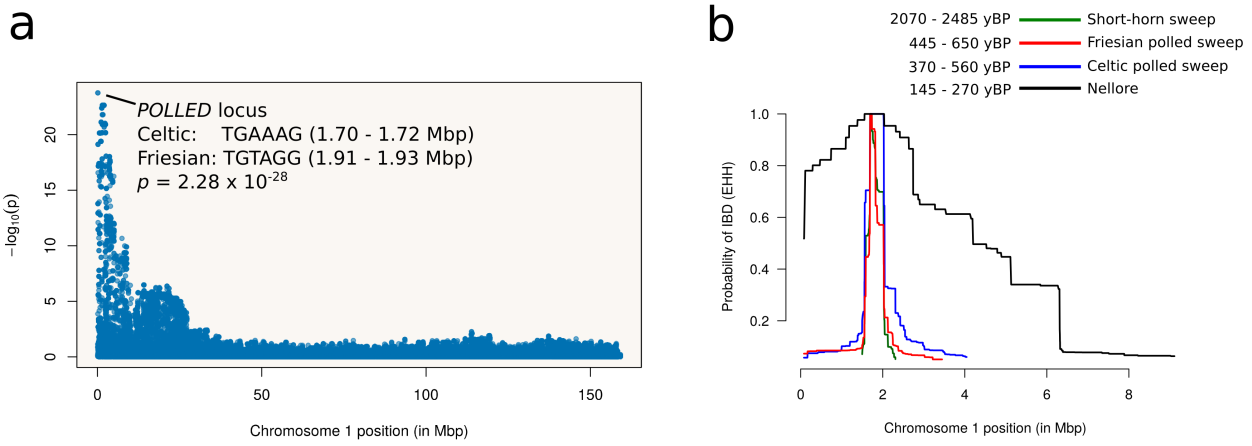

Supplementary Figure 1 Association mapping and coalescence for the *POLLED* locus. (a) Scatterplot showing maximum association (p = 2.28 x 10^{-28}) for horned/polled phenotypes in Nellore cattle mapping to the European *POLLED* selective sweep. The significant haplotypes are of *B. taurus* origin. (b) Extended haplotype homozygosity (EHH). Simultaneous analysis of all Northwestern European breeds reveals that selection for decreased horn size dates back to the Iron Ages. The Celtic and Friesian signatures are much more recent and coalesce to a period close to the selection for *Q*. The polled haplotypes are shown to have been introgressed into Nellore cattle during early imports to Brazil.

Supplementary Table 3 Haplotype frequencies at the Celtic *POLLED* locus in the Bovine HapMap data

Breed	CAGGGA	CAGGAG	CAGAGG	CAGAAG	CGGAGA	CGAAGA	CGAAGG	CGAAAG	TAGGGA	TAGGAG	TAGAGA	TAGAGG	TAGAAG	TGAGGA	TGAAGA	TGAAGG	TGAAAA	TGAAAG
Blonde d'Aquitaine	0.0000	0.0000	0.0000	0.0000	0.0000	0.0000	0.0000	0.0000	0.0000	0.0000	0.0000	0.0000	0.0000	0.0000	0.0000	0.0000	0.0000	1.0000
Normande	0.0000	0.0000	0.0000	0.0000	0.0000	0.0000	0.0000	0.0000	0.0000	0.0385	0.0000	0.0000	0.0000	0.0000	0.0385	0.0385	0.0000	0.8846
Norwegian Red	0.0000	0.0000	0.0000	0.0000	0.0000	0.0000	0.0000	0.0000	0.0000	0.1875	0.0000	0.0000	0.0000	0.0000	0.0000	0.0000	0.0000	0.8125
Red Angus	0.0000	0.0000	0.0000	0.0000	0.0000	0.0000	0.0000	0.0000	0.0000	0.0000	0.0000	0.0000	0.0000	0.0000	0.0000	0.0000	0.0000	1.0000
Holstein	0.0000	0.0833	0.0000	0.0208	0.0000	0.0833	0.0000	0.0000	0.0000	0.0625	0.0000	0.0000	0.0000	0.0000	0.0417	0.0000	0.0000	0.7083
Angus	0.0000	0.0000	0.0000	0.0000	0.0000	0.0000	0.0000	0.0000	0.0000	0.0000	0.0000	0.0000	0.0000	0.0000	0.0000	0.0000	0.0000	1.0000
Limousin	0.0000	0.0000	0.0000	0.0000	0.0000	0.0385	0.0000	0.0000	0.0000	0.0000	0.0000	0.0000	0.0000	0.0000	0.0000	0.0000	0.0000	0.9615
Charolais	0.0000	0.0000	0.0000	0.0000	0.0000	0.0395	0.0000	0.0000	0.0000	0.0000	0.0000	0.0000	0.0000	0.0000	0.0132	0.0000	0.0000	0.9474
Senepol	0.0000	0.0000	0.0000	0.0000	0.0000	0.0000	0.0000	0.0000	0.0000	0.0000	0.0000	0.0000	0.0000	0.0000	0.0000	0.0000	0.0000	1.0000
Wagyu	0.0000	0.0000	0.0000	0.0000	0.0000	0.0735	0.0000	0.0000	0.0000	0.0000	0.0000	0.0000	0.0294	0.0294	0.2206	0.3824	0.0000	0.2647
Hereford	0.0000	0.0000	0.0000	0.0000	0.0000	0.0238	0.0000	0.0000	0.0000	0.0000	0.0000	0.0000	0.0000	0.0000	0.0000	0.0000	0.0000	0.9762
Guernsey	0.0375	0.0000	0.0000	0.0000	0.0000	0.0125	0.0000	0.0500	0.0000	0.0000	0.0000	0.0000	0.0125	0.0000	0.0000	0.0375	0.0000	0.8500
Simmental	0.0000	0.0000	0.0000	0.0000	0.0000	0.0000	0.0000	0.0000	0.0000	0.0000	0.0000	0.0000	0.0000	0.0000	0.0000	0.0000	0.0000	1.0000
Piedmontese	0.0000	0.0000	0.1250	0.0000	0.0208	0.0417	0.0000	0.0833	0.0000	0.0000	0.0000	0.0000	0.0000	0.0000	0.0000	0.0625	0.0000	0.6667
Montbéliard	0.0000	0.0000	0.0000	0.0000	0.0000	0.0000	0.0000	0.0000	0.0000	0.0000	0.0000	0.0000	0.0000	0.0000	0.1000	0.0000	0.0000	0.9000
Jersey	0.0000	0.0000	0.0000	0.0098	0.0000	0.0000	0.0000	0.0000	0.0000	0.0000	0.0000	0.0000	0.0000	0.0000	0.0196	0.0000	0.0000	0.9706
Romagnola	0.0143	0.0000	0.0143	0.0000	0.0143	0.1429	0.0143	0.4286	0.0000	0.0000	0.0000	0.0000	0.0000	0.0000	0.0000	0.1143	0.0000	0.2571
Brown Swiss	0.0000	0.0000	0.0000	0.0000	0.0000	0.1957	0.0000	0.0000	0.0000	0.0000	0.0000	0.0000	0.0000	0.0000	0.0000	0.0109	0.0000	0.7935
N'Dama	0.0833	0.0000	0.3167	0.0000	0.0500	0.0833	0.1333	0.0000	0.0667	0.0000	0.0167	0.0167	0.0000	0.0000	0.0000	0.1833	0.0000	0.0500
Lagunaire	0.0408	0.0000	0.0714	0.0000	0.0816	0.2143	0.1633	0.0204	0.0510	0.0000	0.0000	0.0510	0.0000	0.0000	0.0102	0.0918	0.0000	0.2041
Nellore	0.0000	0.0000	0.2571	0.0000	0.1429	0.0143	0.3286	0.0000	0.1571	0.0000	0.0000	0.0000	0.0000	0.0000	0.0000	0.1000	0.0000	0.0000
Gyr	0.3333	0.0000	0.0000	0.0278	0.0000	0.1667	0.1667	0.0000	0.0833	0.0000	0.0000	0.0000	0.0000	0.0000	0.0000	0.0833	0.0000	0.1389
Beefmaster	0.0070	0.0000	0.0000	0.0000	0.0000	0.0211	0.0000	0.0070	0.0000	0.0000	0.0000	0.0000	0.0352	0.0000	0.0141	0.0000	0.0000	0.9155
Santa Gertrudis	0.0294	0.0000	0.0000	0.0000	0.0000	0.0294	0.0000	0.0294	0.0000	0.0000	0.0000	0.0000	0.0000	0.0000	0.0000	0.0294	0.0000	0.8824
Brahman	0.2292	0.0000	0.0000	0.0000	0.0000	0.2083	0.2708	0.0000	0.0000	0.0000	0.0000	0.0000	0.0000	0.0000	0.0000	0.0000	0.0625	0.2292
Brangus	0.0000	0.0000	0.0000	0.0000	0.0000	0.0000	0.0000	0.0000	0.0000	0.0000	0.0000	0.0000	0.0000	0.0000	0.0000	0.0000	0.0000	1.0000
Sheko	0.0000	0.0000	0.0000	0.0000	0.0000	0.4000	0.4000	0.0000	0.0000	0.0000	0.0000	0.0000	0.0000	0.0000	0.0000	0.0000	0.0000	0.2000
Buffalo	0.0000	0.0000	0.0000	0.0000	0.0000	0.0000	0.0714	0.0000	0.0000	0.0000	0.0000	0.8571	0.0000	0.0000	0.0000	0.0714	0.0000	0.0000
Gaur	0.0000	0.0000	0.0000	0.0000	0.0000	0.0000	0.0000	0.0000	0.0000	0.0000	0.0000	1.0000	0.0000	0.0000	0.0000	0.0000	0.0000	0.0000
Yak	0.0000	0.0000	0.0000	0.0000	0.0000	0.0000	0.0000	0.0000	0.0000	0.0000	1.0000	0.0000	0.0000	0.0000	0.0000	0.0000	0.0000	0.0000

Haplotypes were constructed based on Illumina® BovineHD data of markers BovineHD0100000544 (rs109832358), BovineHD0100000545 (rs135487276), BovineHD0100000546 (rs132949427), BovineHD0100000547 (rs134388759), BovineHD0100000548 (rs109756112) and BovineHD0100000549 (rs133176033).

Supplementary Table 4 Haplotype frequencies at the Friesian *POLLED* locus in the Bovine HapMap data

Breed	CACCAA	CACAGG	CGCCAA	CGCCGG	CGTCGG	CGTAGG	TATCGG	TATAGG	TGCCAG	TGCCGA	TGCCGG	TGTCGG	TGTAGA	TGTAGG
Blonde d'Aquitaine	0.0000	0.0000	0.0000	0.0000	0.0000	0.0000	0.0000	0.0000	0.0000	0.0000	0.0000	0.0000	0.0000	1.0000
Normande	0.0000	0.2000	0.0000	0.0000	0.0000	0.0000	0.0000	0.0000	0.0000	0.0000	0.0000	0.0000	0.0000	0.8000
Norwegian Red	0.0588	0.0000	0.1176	0.0000	0.0294	0.0000	0.0000	0.0294	0.0000	0.0000	0.0000	0.0000	0.0000	0.7647
Red Angus	0.0000	0.0000	0.0000	0.0000	0.0000	0.0000	0.0000	0.0000	0.0000	0.0000	0.0000	0.0000	0.0000	1.0000
Holstein	0.0000	0.0000	0.2887	0.0000	0.0211	0.0282	0.0000	0.0000	0.0000	0.0000	0.0000	0.0000	0.0000	0.6620
Angus	0.0000	0.0000	0.0000	0.0000	0.0000	0.0000	0.0000	0.0000	0.0000	0.0000	0.0000	0.0000	0.0000	1.0000
Limousin	0.0000	0.0000	0.0980	0.0000	0.0000	0.0000	0.0000	0.0000	0.0000	0.0000	0.0000	0.0000	0.0000	0.9020
Charolais	0.0000	0.0000	0.0750	0.0000	0.0250	0.0000	0.0000	0.0000	0.0000	0.0000	0.0000	0.0125	0.0000	0.8875
Senepol	0.0000	0.0000	0.0000	0.0000	0.0000	0.0000	0.0000	0.0000	0.0000	0.0000	0.0000	0.0000	0.0000	1.0000
Wagyu	0.0000	0.0000	0.5000	0.0000	0.0385	0.0385	0.0000	0.0000	0.0000	0.0000	0.0000	0.0385	0.0000	0.3846
Hereford	0.0395	0.0000	0.0132	0.0000	0.0000	0.0000	0.0000	0.0132	0.0000	0.0000	0.0000	0.0000	0.0000	0.9342
Guernsey	0.0238	0.0000	0.1667	0.0000	0.0000	0.0238	0.0000	0.0000	0.0000	0.0000	0.0000	0.0000	0.0000	0.7857
Simmental (Fleckvieh)	0.0000	0.0000	0.0000	0.0000	0.0000	0.0000	0.0000	0.0000	0.0000	0.0000	0.0000	0.0000	0.0000	1.0000
Piedmontese	0.2083	0.0000	0.0833	0.0000	0.1042	0.0000	0.0000	0.0000	0.0000	0.0000	0.0000	0.0000	0.0000	0.6042
Montbéliard	0.0000	0.0000	0.2000	0.0000	0.0000	0.0000	0.0000	0.0000	0.0000	0.0000	0.0000	0.0000	0.0000	0.8000
Jersey	0.0543	0.0000	0.0109	0.0000	0.0109	0.0000	0.0000	0.0109	0.0000	0.0000	0.0000	0.0000	0.0000	0.9130
Romagnola	0.0000	0.0000	0.3088	0.0000	0.0000	0.0000	0.0000	0.0000	0.0000	0.0000	0.0000	0.3382	0.0000	0.3529
Brown Swiss (Braunvieh)	0.0000	0.0000	0.1042	0.0000	0.1875	0.0000	0.0000	0.0000	0.0000	0.0000	0.0000	0.0417	0.0000	0.6667
N'Dama	0.1042	0.0000	0.5208	0.0000	0.0000	0.0000	0.3542	0.0000	0.0000	0.0000	0.0000	0.0000	0.0000	0.0208
Lagunaire	0.0000	0.0000	0.1000	0.0000	0.0000	0.0000	0.7000	0.0000	0.0000	0.0000	0.0000	0.0000	0.0000	0.2000
Nellore	0.0714	0.0000	0.2143	0.0000	0.0000	0.0000	0.0000	0.0000	0.0143	0.0857	0.0571	0.5571	0.0000	0.0000
Gyr	0.1167	0.0000	0.1167	0.0000	0.0000	0.0000	0.0000	0.0000	0.1167	0.0000	0.0333	0.5667	0.0000	0.0500
Beefmaster	0.0000	0.0000	0.0000	0.0000	0.0000	0.0000	0.0000	0.0000	0.1250	0.0000	0.0000	0.0625	0.0000	0.8125
Santa Gertrudis	0.0143	0.0000	0.0143	0.0000	0.1143	0.0000	0.0143	0.0143	0.0143	0.0000	0.0143	0.0000	0.0143	0.7857
Brahman	0.1327	0.0000	0.2041	0.0000	0.0102	0.0000	0.0000	0.0000	0.0306	0.0102	0.0000	0.3878	0.0000	0.2245
Brangus	0.0000	0.0000	0.0385	0.0000	0.0000	0.0000	0.0000	0.0000	0.0000	0.0000	0.0000	0.0000	0.0000	0.9615
Sheko	0.0000	0.0000	0.3611	0.0000	0.0000	0.0000	0.0000	0.0000	0.0000	0.0000	0.0833	0.4167	0.0000	0.1389
Buffalo	0.0000	0.0000	0.0000	0.0000	0.0000	0.0000	0.0000	0.0000	0.0000	0.8571	0.0000	0.1429	0.0000	0.0000
Gaur	0.0000	0.0000	0.0000	0.2500	0.0000	0.0000	0.0000	0.0000	0.0000	0.7500	0.0000	0.0000	0.0000	0.0000
Yak	0.0000	0.0000	0.0000	0.0000	0.0000	0.0000	0.0000	0.0000	0.0000	1.0000	0.0000	0.0000	0.0000	0.0000

Haplotypes were constructed based on Illumina® BovineHD data of markers BovineHD0100000595 (rs136319130), BovineHD0100000596 (rs133617837), BovineHD0100000597 (rs134380776), BovineHD0100000598 (rs137606204), ARS-BFGL-NGS-76349 (rs109797076) and BovineHD0100000599 (rs133424654).

Supplementary Table 5 Haplotype diversity at the CHR14:24973324-25012733 *PLAG1* locus in the Bovine HapMap data

Breed	ATACCT	ATATCT	ATATTC	AGATCC	AGATTC	AGGTTC	GGATTC	GGGTTC (Q)
Blonde d'Aquitaine	0.0000	0.0000	0.0000	0.0000	0.0000	0.0000	0.0000	1.0000
Normande	0.0000	0.0000	0.0000	0.0000	0.0000	0.0000	0.0000	1.0000
Norwegian Red	0.0000	0.0000	0.0000	0.0000	0.0000	0.0000	0.0000	1.0000
Red Angus	0.0000	0.0000	0.0000	0.0000	0.0000	0.0000	0.0000	1.0000
Holstein	0.0070	0.0000	0.0000	0.0000	0.0000	0.0000	0.0000	0.9930
Angus	0.0000	0.0000	0.0000	0.0000	0.0000	0.0000	0.0106	0.9894
Limousin	0.0000	0.0000	0.0000	0.0000	0.0000	0.0000	0.0196	0.9804
Charolais	0.0000	0.0000	0.0000	0.0000	0.0250	0.0000	0.0000	0.9750
Senepol	0.0417	0.0000	0.0000	0.0000	0.0833	0.0000	0.0000	0.8750
Wagyu	0.0000	0.0000	0.0000	0.0000	0.1538	0.0000	0.0000	0.8462
Hereford	0.0000	0.0000	0.0000	0.0000	0.0790	0.0000	0.0790	0.8420
Guernsey	0.0000	0.0000	0.0000	0.0000	0.1670	0.0000	0.1190	0.7140
Simmental (Fleckvieh)	0.0000	0.0000	0.0000	0.0000	0.3500	0.0000	0.1500	0.5000
Piedmontese	0.0000	0.0000	0.0000	0.0000	0.1880	0.0000	0.3330	0.4790
Montbéliard	0.0000	0.0000	0.0000	0.0000	0.4000	0.0000	0.2000	0.4000
Jersey	0.0000	0.0000	0.0000	0.0000	0.3910	0.0000	0.2500	0.3590
Romagnola	0.0000	0.0290	0.0000	0.2940	0.4410	0.0000	0.0590	0.1760
Brown Swiss (Braunvieh)	0.0000	0.0000	0.0000	0.0210	0.2920	0.3960	0.1670	0.1250
N'Dama	0.0000	0.0000	0.0000	0.0000	0.9170	0.0420	0.0210	0.0210
Lagunaire	0.0000	0.0000	0.0000	0.0000	0.3000	0.0000	0.7000	0.0000
Nellore	0.8000	0.1140	0.0140	0.0000	0.0140	0.0000	0.0000	0.0570
Gyr	0.7170	0.2170	0.0330	0.0000	0.0330	0.0000	0.0000	0.0000
Beefmaster	0.1040	0.0000	0.0000	0.0000	0.0630	0.0000	0.0000	0.8330
Santa Gertrudis	0.0430	0.0000	0.0000	0.0000	0.0140	0.0000	0.0140	0.9290
Brahman	0.2960	0.0310	0.0000	0.0000	0.0200	0.0000	0.0000	0.6530
Brangus	0.0000	0.0380	0.0000	0.0000	0.0000	0.0000	0.0000	0.9620
Sheko	0.3890	0.1670	0.0830	0.0000	0.2500	0.0830	0.0000	0.0280
Buffalo	1.0000	0.0000	0.0000	0.0000	0.0000	0.0000	0.0000	0.0000
Gaur	1.0000	0.0000	0.0000	0.0000	0.0000	0.0000	0.0000	0.0000
Yak	1.0000	0.0000	0.0000	0.0000	0.0000	0.0000	0.0000	0.0000

Haplotypes were constructed based on Illumina® BovineHD data of markers BovineHD1400007249 (rs110243083), BovineHD1400007250 (rs136888475), BovineHD1400007253 (rs109636480), BovineHD1400007254 (rs135404594), BovineHD1400007257 (rs134286310) and BovineHD1400007258 (rs135538206).

3. Assessment of genotype imputation based on a reduced set of 24 bulls

We evaluated whether the 24 sequenced Nellore bulls could serve as a reference set for imputation of HD genotypes up to sequence variants. The main idea was that, if accurate predictions of whole genome sequence genotypes could be achieved using these bulls, the haplotype association analysis presented in the main paper could be replaced by direct analysis of sequence variants. Since test animals had only HD data available, extraction of HD genotypes was performed on the 24 sequenced bulls, and test animals had their genotypes reduced to panels of smaller marker density that partially overlapped with the HD array (**Supplementary Table 6**). These panels included the industry's standard Illumina® BovineSNP50 v2 (50K) and two *B. indicus*-specific panels, namely GeneSeek® Genomic Profiler Bos Indicus HD (GGP75Ki) and Illumina® Z-Chip v1 (ZChip). FImpute v2.2 [4] was used to impute genotypes, as it has been shown to yield comparable accuracies with competing algorithms with substantially smaller run times [5,6]. In comparison to Beagle [7], FImpute was found to yield higher accuracies in a wide range of imputation scenarios in this Nellore population [8]. Briefly, FImpute's method is based on a deterministic approach that assumes all animals are related to each other to some degree. Initially, genotypes are predicted using chromosome windows with shared long haplotypes (identical-by-descent segments due to recent ancestors). Then, genotypes are further imputed using shorter windows to capture information from more distant relatives. Accuracy of imputation was measured as the percentage of correctly imputed genotypes. Average accuracies were 81.50 ± 4.21%, 89.10 ± 2.73% and 82.41 ± 4.12% for 50K, GGP75Ki and ZChip, respectively. These results indicated that a larger sample of reference animals would be required for accurate imputation.

Supplementary Table 6 Number of markers per assay used in the imputation analysis

Assay name	Abbreviation	Manifest version	Genome assembly	Total number of markers	Markers in common with HD	Markers in common with HD after filter [a]
Illumina® BovineSNP50 v2	50K	C	UMDv3.1	54,609	49,915	21,239
GeneSeek® Genomic Profiler Bos Indicus HD	GGP75Ki	A	UMDv3.1	74,677	73,959	64.206
Illumina® Z-Chip v1	ZChip	A	UMDv3.1	27,533	27,202	21,333
Illumina® BovineHD	HD	A	UMDv3.1	786,799	786,799	447,617

[a]Autosomal markers that had call rate > 95% and minor allele frequency > 5%.

4. Influence of haplotype size and population structure on association analyses

We re-analyzed the birth weight data in order to assess how population stratification and haplotype size could affect the association results. We first used the same method described in the material and methods with haplotypes of size 1 (equivalent to performing a standard single-marker analysis), 5, 10 and 20, paralleling our original analysis of 6-markers haplotypes. Then, we repeated all analyses considering the following mixed linear model:

$$\mathbf{y} = \mathbf{1}\mu + \mathbf{u} + \mathbf{e}$$

where $\mathbf{y}$ is the vector of dEBVs, $\mathbf{1}$ is a vector of 1s, μ is the intercept, $\mathbf{u}$ is the vector of polygenic effects (i.e., sum of the random effects of genome-wide markers), and $\mathbf{e}$ is the vector of residual effects. The model assumed $\mathbf{y} \sim N(\mathbf{1}\mu, \mathbf{V})$ for $\mathbf{V} = \mathbf{K}\sigma_u^2 + \mathbf{W}\sigma_e^2$, where $\mathbf{K}$ is the genomic relationship matrix (GRM) excluding the tested chromosome, $\mathbf{W}$ is the dEBV weight matrix as described in the material and methods, and σ_u^2 and σ_e^2 are the variance components related to polygenic and residual effects, respectively. Variance components were estimated using restricted maximum likelihood (REML) with the Newton-Raphson algorithm. Similarly to the original analysis, we computed $\alpha_k = (\mathbf{z}_k\mathbf{V}^{-1}\mathbf{z}_k)^{-1}\mathbf{z}_k\mathbf{V}^{-1}(\mathbf{y} - \mathbf{1}\mu)$ and $SE(\alpha_k) = (\mathbf{z}_k\mathbf{V}^{-1}\mathbf{z}_k)^{-1/2}$ for each haplotype k, and the association mapping was based on the two-tailed t-test α_k / $SE(\alpha_k)$. This approach was very similar to the standard Leave-One-Chromosome-Out Mixed Linear Model Association (MLMA-LOCO) method [9], except that our analysis considered haplotypes instead of single-markers, and that heterogeneity of residual variance was explicitly modeled.

We found that association results were robust in respect to variations in haplotype size and presence of population structure (**Supplementary Fig. 2**). Considering no correction for genetic stratification, the most significant associations were found at positions 26007360 ($p = 4.63 \times 10^{-18}$), 24973324-25012733 ($p = 4.77 \times 10^{-17}$), 25692794-25753013 ($p = 6.35 \times 10^{-17}$) and 24406302-24478336 ($p = 2.44 \times 10^{-16}$) for the analyses of haplotypes built over 1, 5, 10 and 20 markers, respectively. In

the case of the mixed model analysis, peaking positions were identified at 24473841 (p = 4.52 x 10^{-9}), 24472819-24478336 (p = 7.18 x 10^{-9}), 25692794-25753013 (p = 1.91 x 10^{-8}) and 24571130-24653754 (p = 4.08 x 10^{-8}) for windows of 1, 5, 10 and 20 markers, respectively. Altogether, these analyses suggested that the causal mutation is most likely located between positions 24406302 and 26007360 bp on chormosome 14. This ~1.6 Mbp window contained the fine-mapped region reported by Boitard *et al*. [10] spanning 24.80 – 25.08 Mbp on CHR14, as well as the candidate quantitative trait nucleotides (QTNs) located between 24.97 and 25.05 Mbp reported by Karim *et al*. [11] (**Supplementary Table 7**). Therefore, we conclude that the ~39.5 kbp haplotype spanning positions 24973324-25012733 bp was a suitable predictor for the *Q* mutation, regardless of the existence of actual physical overlap between the two. As justified in the main paper, inclusion of SNP rs109815800 further improved tagging of *Q* in worldwide cattle breeds, which yielded a final ~42.3 kbp haplotype ranging from 24973324 to 25015640 bp on CHR14 (**Supplementary Table 8**).

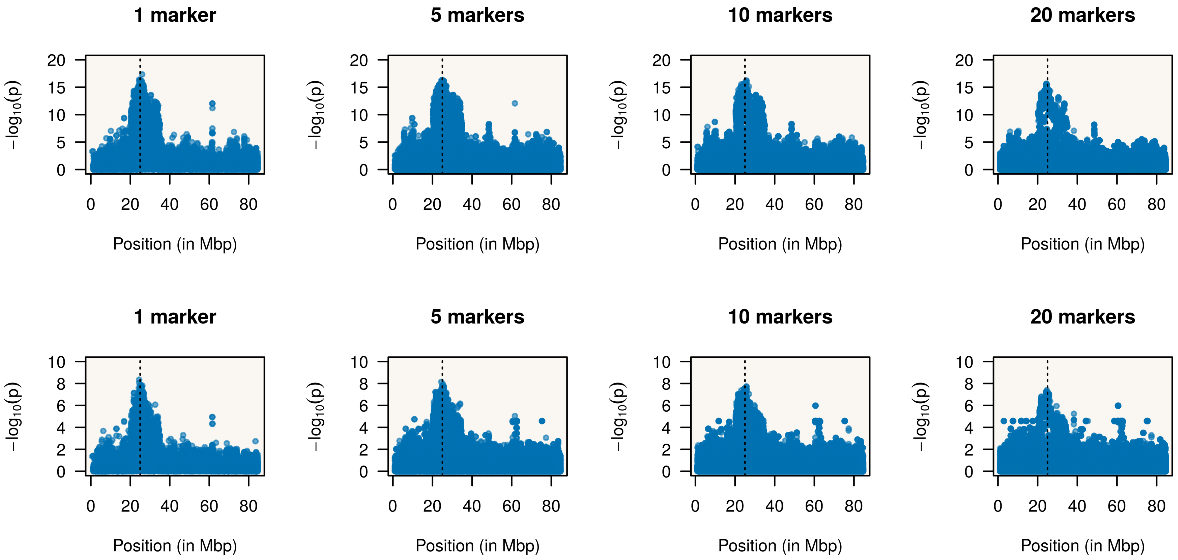

Supplementary Figure 2 Identification of tag haplotypes for the *PLAG1* locus. The upper and lower panels present results for the fixed and mixed model analyses, respectively. Vertical dashed lines indicate the mid-point position (CHR14:25004732) of candidate causal variants reported by Karim *et al*. [11]

Supplementary Table 7 Candidate quantitative trait nucleotides underlying associations on the *PLAG1* chromosomal domain

Variant name	Position	Functional prediction	Included in the Illumina® BovineHD panel	Reference allele	Candidate *q*	Candidate *Q*
rs110092040	24973953	Downstream variant	No	T	C	T
ss319607399	24974221	Downstream variant	No	A	G	A
ss319607400	24974811	Downstream variant	No	A	G	A
rs109231213	25003338	3'-UTR variant	No	C	G	C
ss319607401	25006125	3'-UTR variant	No	T	C	T
rs109815800	25015640	Intron variant	Yes	G	T	G
ss319607405	25052396	Promoter variant	No	$(CCG)_{11}$	$(CCG)_{9}$	$(CCG)_{11}$
ss319607406	25052440	Promoter variant	No	G	A	G

Supplementary Table 8 Haplotype diversity at the CHR14:24973324-25015640 *PLAG1* locus in the Bovine HapMap data

Breed	ATACCTT	ATATCTT	ATATTCT	AGATCCT	AGATTCT	AGATTCG	AGGTTCT	GGATTCT	GGGTTCT	GGGTTCG(*Q*)
Blonde d'Aquitaine	0.0000	0.0000	0.0000	0.0000	0.0000	0.0000	0.0000	0.0000	0.0000	1.0000
Normande	0.0000	0.0000	0.0000	0.0000	0.0000	0.0000	0.0000	0.0000	0.0000	1.0000
Norwegian Red	0.0000	0.0000	0.0000	0.0000	0.0000	0.0000	0.0000	0.0000	0.0000	1.0000
Red Angus	0.0000	0.0000	0.0000	0.0000	0.0000	0.0000	0.0000	0.0000	0.0455	0.9545
Holstein	0.0070	0.0000	0.0000	0.0000	0.0000	0.0000	0.0000	0.0000	0.0000	0.9930
Angus	0.0000	0.0000	0.0000	0.0000	0.0000	0.0000	0.0000	0.0106	0.0000	0.9894
Limousin	0.0000	0.0000	0.0000	0.0000	0.0000	0.0000	0.0000	0.0196	0.0098	0.9706
Charolais	0.0000	0.0000	0.0000	0.0000	0.0250	0.0000	0.0000	0.0000	0.0125	0.9625
Senepol	0.0417	0.0000	0.0000	0.0000	0.0833	0.0000	0.0000	0.0000	0.1250	0.7500
Wagyu	0.0000	0.0000	0.0000	0.0000	0.1538	0.0000	0.0000	0.0000	0.3077	0.5385
Hereford	0.0000	0.0000	0.0000	0.0000	0.0789	0.0000	0.0000	0.0789	0.0526	0.7895
Guernsey	0.0000	0.0000	0.0000	0.0000	0.1667	0.0000	0.0000	0.1190	0.5714	0.1429
Simmental (Fleckvieh)	0.0000	0.0000	0.0000	0.0000	0.3500	0.0000	0.0000	0.1500	0.3000	0.2000
Piedmontese	0.0000	0.0000	0.0000	0.0000	0.1875	0.0000	0.0000	0.3333	0.4792	0.0000
Montbéliard	0.0000	0.0000	0.0000	0.0000	0.4000	0.0000	0.0000	0.2000	0.4000	0.0000
Jersey	0.0000	0.0000	0.0000	0.0000	0.3913	0.0000	0.0000	0.2500	0.2283	0.1304
Romagnola	0.0000	0.0294	0.0000	0.2941	0.4412	0.0000	0.0000	0.0588	0.1618	0.0147
Brown Swiss (Braunvieh)	0.0000	0.0000	0.0000	0.0208	0.2917	0.0000	0.3958	0.1667	0.1250	0.0000
N'Dama	0.0000	0.0000	0.0000	0.0000	0.9167	0.0000	0.0417	0.0208	0.0208	0.0000
Lagunaire	0.0000	0.0000	0.0000	0.0000	0.3000	0.0000	0.0000	0.7000	0.0000	0.0000
Nellore	0.8000	0.1143	0.0143	0.0000	0.0143	0.0000	0.0000	0.0000	0.0143	0.0429
Gyr	0.7167	0.2167	0.0333	0.0000	0.0333	0.0000	0.0000	0.0000	0.0000	0.0000
Beefmaster	0.1042	0.0000	0.0000	0.0000	0.0625	0.0000	0.0000	0.0000	0.0000	0.8333
Santa Gertrudis	0.0429	0.0000	0.0000	0.0000	0.0000	0.0143	0.0000	0.0143	0.0143	0.9143
Brahman	0.2959	0.0306	0.0000	0.0000	0.0204	0.0000	0.0000	0.0000	0.0204	0.6327
Brangus	0.0000	0.0385	0.0000	0.0000	0.0000	0.0000	0.0000	0.0000	0.0385	0.9231
Sheko	0.3889	0.1667	0.0833	0.0000	0.2500	0.0000	0.0833	0.0000	0.0278	0.0000
Buffalo	1.0000	0.0000	0.0000	0.0000	0.0000	0.0000	0.0000	0.0000	0.0000	0.0000
Gaur	1.0000	0.0000	0.0000	0.0000	0.0000	0.0000	0.0000	0.0000	0.0000	0.0000
Yak	1.0000	0.0000	0.0000	0.0000	0.0000	0.0000	0.0000	0.0000	0.0000	0.0000

Haplotypes were constructed based on Illumina® BovineHD data of markers BovineHD1400007249 (rs110243083), BovineHD1400007250 (rs136888475), BovineHD1400007253 (rs109636480), BovineHD1400007254 (rs135404594), BovineHD1400007257 (rs134286310), BovineHD1400007258 (rs135538206) and BovineHD1400007259 (rs109815800).

5. Influence of local recombination rates on the estimates of age of selection

Since the age of the *Q* selective sweep was initially dated based on average recombination rates, we re-analyzed the data using sex-specific high resolution recombination maps reported by Ma *et al.* [12] (available at: http://datadryad.org/resource/doi:10.5061/dryad.q2q84). These maps were generated from a large pedigree of 186,927 three-generation families and built over ~8.5 million observed recombination events. Genetic distances were first computed using Haldene's map function [13], then missing values were linearly interpolated. As shown in **Supplementary Table 9**, the use of local or average recombination rates provided very similar results.

Supplementary Table 9 Estimates of 95% confidence intervals for the age of the *Q* selective sweep using different genetic maps

Population	Average (1.23 Mbp ~ 1 cM)	Female[a]	Male[a]
European *B. taurus*	305 - 475	250 - 410	250 - 405
Japanese *B. taurus*	90 - 195	100 - 205	100 - 205
Brahman	75 - 170	70 - 165	70 - 165
Nellore	30 - 100	30 - 95	30 - 100

[a]Sex-specific genetic maps were generated from recombination rates reported by Ma *et al*. [12].

References

1. Medugorac, I. *et al*. Bovine polledness - an autosomal dominant trait with allelic heterogeneity. *PLoS ONE*. **7**, e39477 (2012).

2. Allais-Bonnet, A. *et al*. Novel insights into the bovine polled phenotype and horn ontogenesis in Bovidae. *PLoS ONE.* **8**, e63512 (2013).

3. Utsunomiya, A. T. H. *et al*. Revealing misassembled segments in the bovine reference genome by high resolution linkage disequilibrium scan. *BMC Genom*. **17**, 705 (2016).

4. Sargolzaei, M., Chesnais, J. P. & Schenkel, F. S. A new approach for efficient genotype imputation using information from relatives. *BMC Genom*. **15**, 478 (2014).

5. Piccoli, M. L., Braccini, J., Cardoso, F. F., Sargolzaei, M., Larmer, S. G. & Schenkel, F. S. Accuracy of genome-wide imputation in Braford and Hereford beef cattle. *BMC Genet*. **15**, 157 (2014).

6. Ventura, R. V., Lu, D., Schenkel, F. S. & Wang, Z. Impact of reference population on accuracy of imputation from 6K to 50K single nucleotide polymorphism chips in purebred and crossbreed beef cattle. *J Anim Sci*. **92**, 1433-1444 (2014).

7. Browning, B. L. & Browning, S. R. A unified approach to genotype imputation and haplotype-phase inference for large data sets of trios and unrelated individuals. *Am J Hum Genet*. **84**, 210-223 (2008).

8. Carvalheiro, R. *et al*. Accuracy of genotype imputation in Nelore cattle. *Genet Sel Evol*. **46**, 69 (2014).

9. Yang, J., Zaitlen, N. A., Goddard, M. E., Visscher, P. M. & Price, A. L. Advantages and pitfalls in the application of mixed-model association methods. *Nat Genet*. **46**, 100-106 (2014).

10. Boitard, S., Boussaha, M., Capitan, A., Rocha, D. & Servin, B. Uncovering adaptation from sequence data: Lessons from genome resequencing of four cattle breeds. *Genetics*. **203**, 433-450 (2016).

11. Karim, L. *et al*. Variants modulating the expression of a chromosome domain encompassing *PLAG1* influence bovine stature. *Nat Genet*. **43**, 405-413 (2011).

12. Ma, L. *et al*. Cattle Sex-Specific Recombination and Genetic Control from a Large Pedigree Analysis. *PLoS Genet*. **11**, e1005387 (2015).

13. Haldane, J. B. S. The combination of linkage values and the calculation of distances between the loci of linked factors. *J Genet*. **8**, 299-309 (1919).
